# Modeling Coevolution between Language and Memory Capacity during Language Origin

**DOI:** 10.1371/journal.pone.0142281

**Published:** 2015-11-06

**Authors:** Tao Gong, Lan Shuai

**Affiliations:** Haskins Laboratories, New Haven, Connecticut, United States of America; University of Akron, UNITED STATES

## Abstract

Memory is essential to many cognitive tasks including language. Apart from empirical studies of memory effects on language acquisition and use, there lack sufficient evolutionary explorations on whether a high level of memory capacity is prerequisite for language and whether language origin could influence memory capacity. In line with evolutionary theories that natural selection refined language-related cognitive abilities, we advocated a coevolution scenario between language and memory capacity, which incorporated the genetic transmission of individual memory capacity, cultural transmission of idiolects, and natural and cultural selections on individual reproduction and language teaching. To illustrate the coevolution dynamics, we adopted a multi-agent computational model simulating the emergence of lexical items and simple syntax through iterated communications. Simulations showed that: along with the origin of a communal language, an initially-low memory capacity for acquired linguistic knowledge was boosted; and such coherent increase in linguistic understandability and memory capacities reflected a language-memory coevolution; and such coevolution stopped till memory capacities became sufficient for language communications. Statistical analyses revealed that the coevolution was realized mainly by natural selection based on individual communicative success in cultural transmissions. This work elaborated the biology-culture parallelism of language evolution, demonstrated the driving force of culturally-constituted factors for natural selection of individual cognitive abilities, and suggested that the degree difference in language-related cognitive abilities between humans and nonhuman animals could result from a coevolution with language.

## Introduction

### Perspectives and approaches on language evolution and human cognition

Origins and evolution of human language have recently obtained a wide scope of academic interests [1–4]; in particular, origins and evolution of *language faculty* (the set of competencies for grasping and using any natural language [5]) have attracted not only linguists but also scholars from other relevant disciplines [6–10]. Per this topic, the ‘saltational’ views [11,12] highlight the dissociation of language from cognition, and ascribe the uniqueness of language to catastrophic mutations [13] or human/language-specific mechanisms (e.g., recursion [5,14]). By contrast, the ‘gradualist’ views stress that the components of the language faculty must have derived from domain-general abilities shared by humans and other animals [6,15,16], via a series of Darwinian natural selection [17,18] or *coevolution* (the process whereby organisms adjust selective pressures on others, and meanwhile, receive influential feedback on selections toward their own [19]) [20,21] in a *cultural niche* [22–24].

Among the ‘gradualist’ views, some argue that sufficiently-high degrees of domain-general, socio-cognitive abilities in humans must be prerequisite for language and human communication [25–27]; in other words, a set of fully-fledged cognitive abilities constitute a “language-ready” brain [10]. By contrast, others advocate that along with language evolution, communicative success in the human cultural niche serves as the driving force for selecting language-related cognitive abilities in humans [6,17,21,23,28]; that is to say, the domain-general abilities for language might not have been fully-fledged in humans at the time of language origin, and the distinctive degree differences in those abilities between humans and other species could result from subsequent selections on those abilities along with language evolution. Regarding the mechanisms refining language-related cognitive abilities, some theories suggest that natural selection must play a leading role in choosing capable language users for reproduction and spreading their advanced language-related cognitive abilities [17,23,29], whereas other hypotheses claim that cultural evolution alone can account for the recruitment of cognitive abilities and the origin of syntax [30,31].

At least two aspects of evidence are needed to evaluate these contradictory perspectives: the evidence that development of idiolects or understandability of communal language correlates with socio-cognitive abilities; and the evidence that communicative success can trigger necessary adjustment on the degrees of related socio-cognitive abilities among language users. Psychological, genetic, and neuroscience studies on subjects exhibiting a variety of impairments on both cognitive and linguistic abilities have consistently demonstrated that a continuum of cognitive abilities resulting from certain neural or genetic influence could cause apparent order-disorder dichotomy in language performance [32–35]. Comparative research has also revealed that compared to humans many nonhuman species possess relatively-low degrees of certain socio-cognitive abilities and lack communication systems as sophisticated as language (e.g., [8,9,16]).

From an evolutionary perspective, the approaches based on human adults, pre-language children, and contemporary social animals offer no clues to the second aspect of evidence, because apart from the ultimate outcome (having language and fully-fledged cognition in humans, but not in nonhuman species), they could not reflect any intermediate evolutionary stages of language and human cognition. Relying solely on comparative findings between humans and nonhuman species or between normal and deficit children may even lead to presumptuous claims that high levels of certain abilities (e.g., shared intentionality [25]) in humans are prerequisite for language acquisition and origin.

Noting these, scholars start to make use of other approaches to verify the evolutionary relation between language and cognition. A recently-adopted approach is computer simulation. It allows for manipulating individual activities and socio-cultural environments, thus offering a systematic and quantitative analysis of the theoretical claims about language evolution [36,37]. In the past few decades, many computational models have been developed to address issues concerning language and its evolution. For example, Batali proposed an artificial neural network model simulating the origin of simple grammar [38], Kirby designed an iterated learning model tracing the origin of a compositional language via cultural transmission between generations of artificial language users [39], and Steels and colleagues developed a series of language game models illustrating the cultural evolution of complex grammatical constructions [31,40]. Few of these models have targeted the evolutionary relations between language and cognitive abilities in humans. For example, Gong and Shuai have simulated a coevolution between language and *joint attention* (the socio-cognitive ability for establishing common ground in interactive activities [25]) [41]. This study provided supportive evidence for the coevolution between language and joint attention: the degree of joint attention appears correlated with linguistic mutual understandability; and communicative success can gradually boost an initially-low level of joint attention, and ratchet it to a sufficient level for the origin of a communal language with high understandability.

Apart from joint attention, linguistic communication also relies on other socio-cognitive abilities. A comprehensive validation of the possible coevolution between language and cognition calls for additional investigations on the possible coevolution between language and those abilities.

### Coevolution scenario between language and memory capacity

*Memory*, as cognitive processes to encode, store, or retrieve information, is one of fundamental requisites for communicative activities. In general, the memory system in humans consists of *short-term memory* (STM) and *long-term memory* (LTM) [42]. STM involves central executive (supervising related components or processes), phonological loop (recording sound or phonological stimuli), visuospatial sketchpad (holding visual or spatial information), and episodic buffer (integrating cross-domain information to form visual, spatial, or verbal units) [43]. Apart from recording environmental information, STM also serves as *working memory* (WM) for cognitive activities such as sensing, reasoning, and learning [44,45]. LTM includes implicit memory and declarative memory. Declarative memory can be divided into episodic and semantic memories, both storing factual information, general knowledge, and personal experience [46] that are extracted from the information in STM [47]. STM is subject to disruption, whereas LTM, once consolidated, is stable. Information in STM also differs from that in LTM in terms of degrees of persistence and abstractness.

Language is the primary means of exchanging conceptual and semantic information in humans. The acquisition, processing, and evolution of language correlate with the memory system in many aspects. First, the memory components participate in many facets of language use. For example, phonological loop and visuospatial sketchpad help record meaning-utterance mappings recently obtained in communications, which constitute individual linguistic experience [48,49]. Episodic and semantic memories help store acquired knowledge from individual experience for future communications. In addition, comparative evidence has revealed that STMs in humans and other animals have similar constraints [50,51], whereas human LTM appears superior in the domain of language use. For example, nonhuman animals exhibit a low degree (in terms of duration and type of stored items) of episodic-like memory and such memory is used predominately for memorizing information about food or shelter locations, whereas the episodic and semantic memories in humans can not only store but also construct concrete and abstract knowledge of individual items, autobiographical events, and their correlations [16]. Furthermore, psycholinguistic studies have revealed many memory constraints on language use (e.g., [52,53]) and a variety of memory deficits that could influence language acquisition and use (e.g., the short-term or working memory deficit [54], the phonological memory deficit [55], and the procedural or declarative memory deficit [56]).

Apart from psychological and comparative studies, there have been theoretical discussions highlighting the necessity of memory for language evolution (e.g. [57–59]). However, many of these theories (e.g. [9,10]) directly presume a high degree of memory for language to emerge, without addressing evolutionary questions such as how memory capacities for language use are gradually formed and how memory and language influence each other during language evolution. In addition, due to lacking quantitative approaches, many theoretical discussions are restricted at a qualitative level, unable to illustrate the quantitative dynamics of the evolutions of language and memory system. Answers to these issues can shed important light on our understanding of the evolutionary relations between language and cognition [53].

In line with the coevolution hypothesis between language and cognition and inspired by the early simulation of the coevolution between language and joint attention, we propose a coevolution scenario for the development of memory capacity for language use during language origin. For the sake of simplicity, our scenario focuses in particular on the evolution of LTM, but involves both STM and LTM. It assumes that early hominins could temporarily record, in their STM units, meaning-utterance mappings obtained in recent communications with other individuals. Without preliminary linguistic knowledge, the meanings of such mappings were obtained mainly from nonlinguistic environmental cues. Given many of such linguistic instances, based on general learning mechanisms, early hominins could extract recurrent matching patterns between partial utterances and meanings from stored instances in their STMs, and allocate LTM units to store these patterns as acquired linguistic knowledge. At this stage, difference in individual LTM capacities could result in storing various amounts of linguistic knowledge, and thereby render distinct levels of communicative success among individuals. Accordingly, along with the origin of a communal language, natural and/or cultural selections could take effect to adjust individual LTM capacities.

### Selecting models to simulate language-memory coevolution

To evaluate the coevolution scenario using computer simulation, we need a language origin model that matches at least four criteria. Although there have been many multi-agent models implementing various aspects of coevolution during language evolution, these criteria make many of them not suitable to illustrate the language-memory coevolution.

First of all, the expected language origin model needs to explicitly separate the memory system into STM for recording exchanged linguistic instances and LTM for storing acquired knowledge. As stated above, STM and LTM differ in many aspects, and abstract knowledge in LTM (e.g., linguistic knowledge of lexical items and syntax) is often extracted from concrete examples in STM (e.g., utterances formed by linguistic knowledge). Such separation allows an exploration of the evolutions between language and a particular type of memory (e.g., LTM in our study). By contrast, many lexical evolution models [30] (e.g., the naming game [60] or the category game [61]) did not explicitly separate LTM from STM, and many syntactic evolution models (e.g., the iterated learning model [39]) lumped both concrete (holistic utterances exchanged) and abstract information (compositional knowledge) together in the same unlimited memory system.

Second, artificial individuals in the expected model can apply instance-based learning mechanisms to acquire linguistic knowledge, and the parameters controlling the learning mechanisms are relatively independent from those manipulating memory capacities. This allows studying the evolution of memory capacities under a general setting, independent of the effects of learning mechanisms or other factors.

Third, the expected model should contain informative indices tracing not only the origin of a communal language consisting of common knowledge shared by individuals, but also the adjustment on individual memory capacities. This helps illustrate the possible coevolution and discuss the respective and collective roles of natural and cultural selections in the coevolution. The iterated learning model [39] and its extensions (e.g. [62]) entangled lexical and syntactic knowledge, and the setting of single-individual generations made them hard to disentangle cultural and natural selections. In addition, due to the focus on the evolutions of grammatical constructions, artificial agents in many language game models (e.g. [40]) directly recruited relevant learning mechanisms to handle language materials, without manipulating their levels. This made these models unable to trace the coevolution between language and cognitive abilities during language origin, which manifests primarily in terms of level change in relevant cognitive abilities and linguistic understandability.

Fourth, the acquired linguistic knowledge in the expected model should not be limited to lexical items. This criterion makes sure that the emergent language in the model is distinct from lexicon-like communication systems as in some nonhuman species, and avoids showing a superficial correlation between language and memory (i.e., an increasing number of lexical items certainly requires an increase in memory capacity for storage). The outcome of the coevolution between language and memory capacity is not merely an increase in memory capacity for keeping relevant linguistic knowledge, but also a transition of linguistic knowledge to accommodate limited capacity. Such transition is collectively induced by individual learning mechanisms and acquired linguistic knowledge. It resolves the conflict between unlimited expressions and limited storing capacities, and allows language users to encode an unlimited number of meanings using a limited number of expressions. It is not evident in most animal communication systems, and cannot be observed in lexical evolution models without grammar learning mechanisms.

Considering these, we adopt the lexicon-syntax coevolution model [63,64] in our study of language-memory coevolution. The model was first designed to simulate a collective acquisition of lexical items and constituent word orders out of a holistic protolanguage in a multi-agent population. In the model, artificial agents are equipped with both STM units to store exchanged meaning-utterance instances, and LTM units to record lexical and syntactic knowledge extracted from these instances. In addition, they apply domain-general abilities such as pattern extraction and sequential learning to acquire lexical and syntactic knowledge. These abilities resemble those used by language learning children [65] or early hominins [8,66]. The initial holistic language resembles the hypothesized protolanguage of early hominids, and the origin of a compositional language comprising a common lexicon and consistent word orders follows some language origin scenarios [9,66,67]. Note that our study does not intend to validate any language origin scenarios; models following other scenarios are also acceptable provided that they match the above-mentioned four criteria.

Based on the theoretical framework adapted from the early simulation of the coevolution between language and joint attention [41], our simulations reveal an inherent coevolution between individual LTM capacity and the degree of mutual understandability of the emergent language: if the initial LTM capacity appears to be smaller than the full expressivity of the language, along with the origin of a communal language, the LTM capacity increases to better record acquired linguistic knowledge; and after reaching a sufficient degree, the LTM capacity will not change greatly. Statistical analyses further show that such coevolution is driven by communicative success during cultural transmissions and achieved mainly by natural selection.

These results provide supporting evidence for the coevolution hypotheses between language and related cognitive abilities, and suggest an alternative evolutionary trajectory, contrary to the saltational or prerequisite views [11,12,25], leading to the degree difference in language-related abilities between humans and nonhuman species. They also highlight the imperativeness of both biological and cultural transmissions for the coevolution, and trigger reconsideration on the clear-cut distinction between biological and cultural evolutions (e.g. [10]) and any biased views stressing the dominant roles of natural selection (e.g. [18]) or cultural selection (e.g. [31]) in language evolution.

In the following sections, we first introduce the language origin model, and then describe the theoretical framework and simulation setup. After that, we illustrate the results of five sets of simulations with and without natural and/or cultural selections. Finally, we summarize the language-memory coevolution manifest in these simulations, and discuss relevant challenges to the coevolution hypothesis between language and cognition.

## Language Origin Model

### Language and individuals

The model encodes language as *meaning-utterance mappings* (*M-U mappings*). Artificial individuals share a semantic space that contains a fixed number of *integrated meanings*, each having a “*predicate*〈*agent*〉” or “*predicate*〈*agent*, *patient*〉” structure. These semantic structures are most frequent in world languages. Here, *predicate*, *agent*, and *patient* are thematic roles. *Predicates* refer to actions that individuals can conceptualize (e.g., “to run” or “to chase”), and *arguments* entities on or by which actions are performed (e.g., “fox” or “tiger”). Some predicates each take a single argument, e.g., “run〈tiger〉” (“a tiger is running”); others take two, e.g., “chase〈tiger, fox〉” (“a tiger is chasing a fox”), where the first constituent within〈〉, “tiger”, denotes the *agent* (action instigator) of the predicate “chase”, and the second, “fox”, the *patient* (entity that undergoes the action) of the predicate. In this model, *agent* and *patient* constituents are animate and chosen from the same set. Comprehension needs both lexical knowledge to interpret semantic constituents and syntactic knowledge to clarify thematic roles (who is *agent* and who is *patient*). For the sake of simplicity, integrated meanings having identical *agent* and *patient* constituents (e.g., “fight〈fox, fox〉”) are excluded.

We define the semantic space by specifying the respective numbers of semantic constituents being *agent*, *patient*, single- and double-argument *predicate*. For example, if there are 4 *agent* or *patient* constituents, 4 single- and 4 double-argument *predicate* constituents, the semantic space will contain 64 integrated meanings, 16 (4×4) of which are “*predicate*〈*agent*〉” meanings and 48 (4×4×(4–1)) are “*predicate*〈*agent*, *patient*〉” meanings. If the numbers of *agent*, *patient*, and *predicate* constituents are 5, there will be 125 (5×5+5×5×(5–1)) integrated meanings; and if the numbers are 6, there will be 216 (6×6+6×6×(6–1)) integrated meanings in the semantic space.

Integrated meanings are encoded into *utterances*, each consisting of a string of syllables chosen from a fixed signalling space. An utterance encoding an integrated meaning can be segmented into subparts, each mapping one or two constituents; and subparts can combine to form an integrated meaning.

Individuals are simulated as *artificial agents*, who, based on their learning mechanisms, can acquire linguistic knowledge from M-U mappings obtained in recent communications, and apply acquired knowledge to produce utterances encoding integrated meanings and to comprehend utterances into integrated meanings in communications with other agents.

### Linguistic knowledge

**Fig 1:**
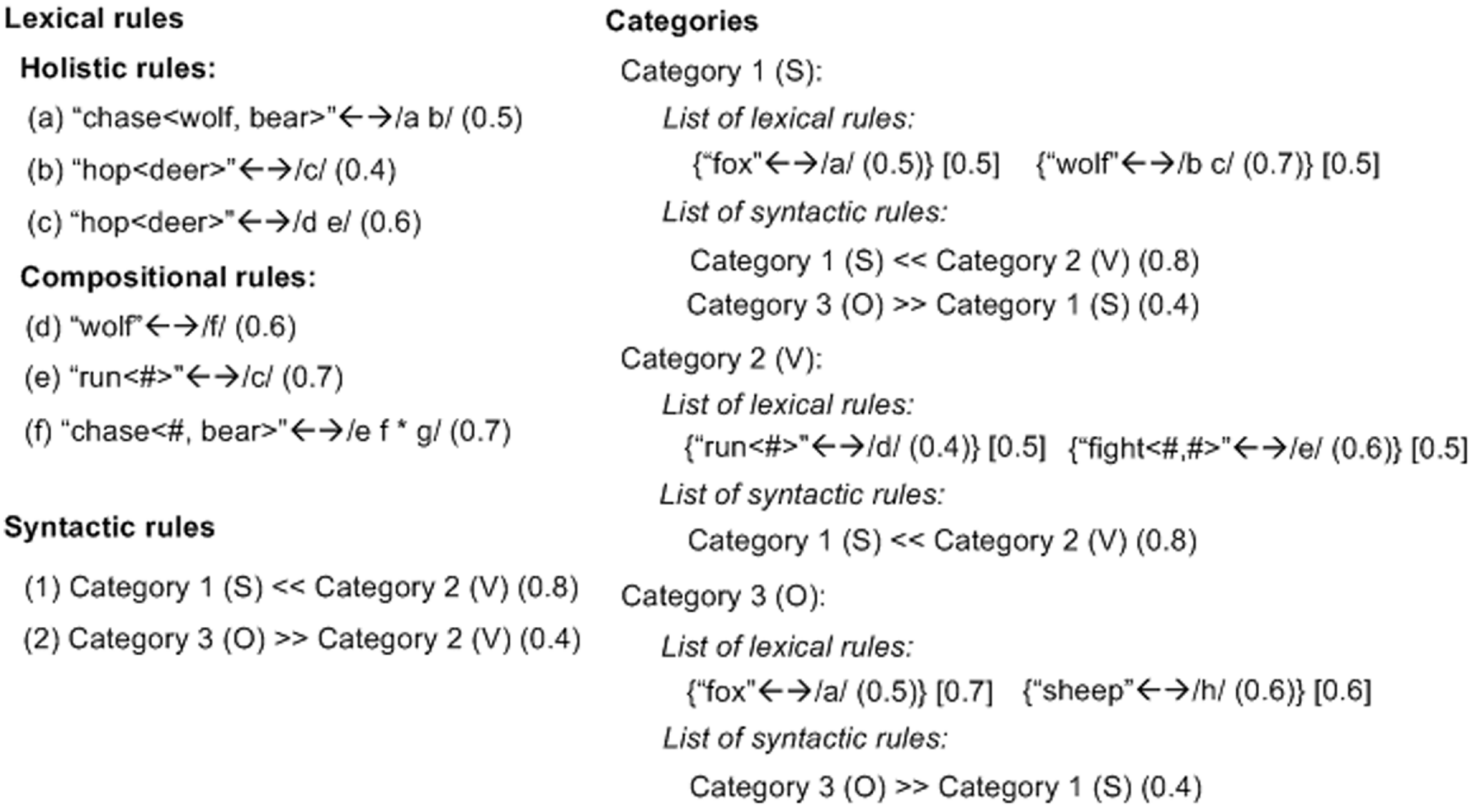
Examples of lexical rules, syntactic rules, and categories. “#” denotes unspecified constituents, and “*” unspecified syllable(s). S, V, and O are syntactic roles of categories. Numbers in () denote rule strengths, and those in [] association weights. “<<” denotes the local order *before*, and “>>” *after*. Compositional rules can combine, if specifying each constituent in an integrated meaning exactly once, e.g., rules (c) and (d) can combine to encode “chase〈wolf, bear〉” with /ehfg/. Lexical and syntactic knowledge collectively encode integrated meanings, e.g., to express “fight〈wolf, fox〉” using the lexical rules in the S, V, and O categories and the syntactic rules SV and SO, the resulting sentence is /bcea/ or /bcae/, following SVO or SOV.

The model simulates three types of simple linguistic knowledge, namely lexicon, syntax, and categories (see Fig 1 for examples). An individual’s lexicon consists of a number of *lexical rules*. Some lexical rules are *holistic*, each mapping an integrated meaning onto an utterance, e.g., “run〈tiger〉” ↔ /abcd/ indicates that the integrated meaning “run〈tiger〉” can be encoded by the utterance /abcd/ as a whole, and also that /abcd/ can be decoded as “run〈tiger〉”; other lexical rules are *compositional*, each mapping one or two constituent(s) onto a subpart of an utterance, e.g., “fox” ↔ /ef/ or “chase〈wolf, #〉” ↔ /gh*i/ (“#” denotes an unspecified constituent, and “*” unspecified syllable(s)).

A *syntactic rule* encodes a local order (*before* or *after*, not necessarily immediately before or after) between two lexical rules or two sets (categories) of lexical rules.

Categories allow syntactic rules acquired from some lexical items to be applied to other lexical items having the same thematic role. A *category* contains a list of lexical rules and a list of syntactic rules. For the sake of simplicity, we simulate a nominative-accusative language and exclude passive voice. Accordingly, a category associating lexical rules encoding *agent* constituents can also be denoted as a subject (S) category, since *agent* corresponds to S. Similarly, *patient* corresponds to object (O), and *predicate* to verb (V). Then, a local order between two categories can be denoted by their syntactic roles, e.g., an order *before* between an S and a V category can be denoted by SV. S, V and O categories are among the most frequent linguistic categories in world languages.

To implement rule competition and forgetting, each lexical or syntactic rule is assigned a *strength* (within [0.0, 1.0], a newly-acquired rule has a strength 0.5), denoting the probability of successfully using it during communications. A compositional rule also has *association weights* (also within [0.0, 1.0], a newly-formed association has a weight 0.5) to categories involving it, indicating the probability of successfully applying the syntactic rules in those categories on it during communications.

### Individual memory system

Each agent uses STM to store M-U mappings obtained in recent communications in which this agent was the listener, and LTM to record acquired linguistic knowledge (lexical rules, syntactic rules, and categories) extracted from recorded instances in STM. One STM unit stores one M-U mapping, and one LTM unit one piece of linguistic rule.

Both STM and LTM have fixed capacities, but the contents in them are updated differently during communications. When STM is *full* (every unit is occupied by a mapping), a newly-obtained mapping replaces the oldest one. By contrast, when LTM is *full* (every LTM unit is taken by a linguistic rule), a newly-acquired rule replaces the one having the lowest rule strength. In addition, due to unsuccessful use and regular forgetting, some rules may have zero or negative rule strengths or associate weights to some categories, and such rules are immediately discarded from LTM.

Among the three types of linguistic rules, lexical rules are the most fundamental. Other types of rules are built upon lexical rules; syntactic rules record local orders between lexical rules, and categories specify which syntactic rules can be applied to which lexical rules. We limit the current study to the possible coevolution between language and the LTM capacity for lexical rules. Accordingly, we fix the STM capacity for M-U mappings and the LTM capacity for the other types of linguistic rules at *reasonable* levels, such that these capacities are neither too small to store sufficient M-U mappings and syntactic or categorical knowledge, nor too big such that they can unrealistically record every piece of linguistic instance or knowledge.

### Language learning mechanisms

**Fig 2:**
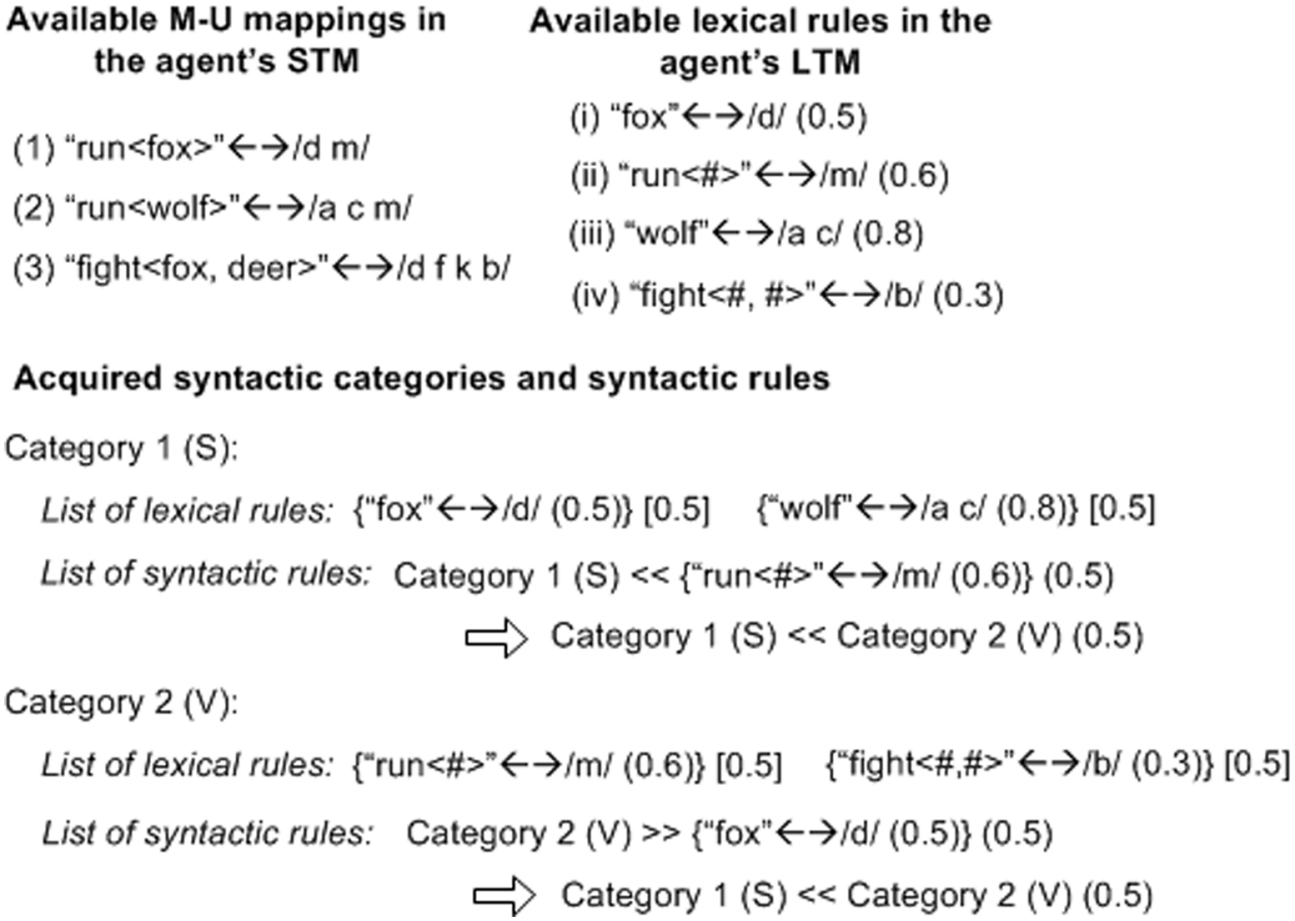
Examples of acquisition of syntactic categories and syntactic rules. M-U mappings are itemized by Arabic numbers, and lexical rules by Roman numbers.

Agents use general learning mechanisms to acquire linguistic rules (see [63,64] for details). Lexical rules are acquired from semantic constituent(s) and utterance syllable(s) that appear repetitively in two or more M-U mappings stored in the STM. New mappings, before being inserted to the STM, are contrasted with those already existent. For example, see Fig 2, by comparing the mappings “run〈fox〉”↔/dm/ and “run〈wolf〉”↔/acm/, an agent can detect the recurrent patterns “run〈#〉” and /m/. If there is no lexical rule recording this mapping, the agent will create a lexical rule “run〈#〉” ↔ /m/, and put it in the LTM.

Categories and syntactic rules are acquired based on the thematic roles of lexical rules and order relations of their utterances in M-U mappings stored in the STM. If an agent notices that in some mappings, the utterances of two or more lexical rules having the same thematic role appear consistently before (or after) the utterance of another lexical rule (or the utterances of another set (category) of lexical rules all having the same thematic roles), the agent can associate these lexical rules into a category having the corresponding syntactic role, create a syntactic rule to record the local order with respect to the other lexical rule(s), and put the syntactic rule to the same category. The category and syntactic rule are stored respectively in the corresponding LTMs for such knowledge.

For example, see Fig 2, evident in mappings (1) and (2), both syllables /d/ of rule (i) and /ac/ of rule (iii) precede /m/ of rule (ii). Since “wolf” and “fox” are both *agents* in these meanings, rules (i) and (iii) can be associated into an S category (Category 1), and the order *before* between these two rules and rule (ii) can be acquired as a syntactic rule. Similarly, in mappings (1) and (3), /m/ of rule (ii) and /b/ of rule (iv) follow /d/ of rule (i), thus inducing a V category (Category 2) associating rules (ii) and (iv) and a syntactic rule *after*. Now, since Categories 1 and 2 respectively associate rules (i) and (iii) as well as rules (ii) and (iv), the two syntactic rules are updated as “Category 1 (S) << Category 2 (V)” (or simply SV), i.e., the syllables of the lexical rules in the S category precede those in the V category.

In this way, agents gradually construct categories linking different lexical rules with different local orders, and merge categories having identical syntactic roles. Finally, all lexical rules encoding semantic constituents having the same thematic roles can be largely associated into the same categories having the corresponding syntactic roles, and the local orders among these categories can form a consistent global order to regulate the lexical rules from these categories in utterance.

These learning mechanisms are in line with the item-based [65], connectionist [68,69], exemplar-based [70] and other usage-based [71,72] accounts of language acquisition, all stressing that language is acquired and processed in a piecemeal and bottom-up fashion.

### Communication

A linguistic communication involves two agents (a speaker and a listener), who perform a number of *utterance exchange*. Each exchange proceeds in three steps: speaker’s production, listener’s comprehension, and update of both agents.

In production, the speaker first selects randomly an integrated meaning from the semantic space. Then, it chooses some of its lexical, syntactic, and category rules in its LTM to form one or several candidate sets for production, each offering an utterance to encode the chosen meaning. After that, the speaker calculates the combined strength of each set, chooses the set having the highest combined strength, builds the utterance accordingly, and transmits the utterance to the listener. The combined strength is calculated as the sum of the lexical contribution (average strength of the lexical rules in this set) and syntactic contribution (average product of the strengths of the syntactic rules regulating the lexical rules and the association weights of those lexical rules to the categories in this set):

Combined Strength=Avg(str(LexRule(s)))+Avg(aso(Cats)×str(SynRule(s))) (1)

Where *Avg* means taking average, *str* means rule strength, *aso* means association weights, and *LexRule(s)*, *SynRule(s)* and *Cats* are lexical rules, syntactic rules and categories. For example, see Fig 1, the three categories, three lexical rules encoding “wolf”, “fox”, and “fight〈#, #〉”, and two syntactic rules SV and SO in those categories collectively form a candidate set to encode “fight〈wolf, fox〉”. The combined strength of this set is 0.98, in which the lexical contribution is 0.6 ((0.7+0.6+0.5)/3) and the syntactic contribution is 0.38 ((0.8×(0.7+0.6)/2+0.4×(0.7+0.5)/2)/2).

If lacking enough rules to encode the chosen meaning, the speaker either makes no production or occasionally (under *the creation rate*) creates a holistic rule to encode the whole meaning, puts this rule in its LTM, and transmits the utterance of this rule to the listener.

In comprehension, the listener receives the utterance produced by the speaker and an *environmental cue*. The cue, as non-linguistic information, contains an integrated meaning plus a *cue strength*. Cues are unreliable (not always containing the speaker’s intended meaning); otherwise, linguistic communications would become unnecessary, since meanings in exchanged utterances can be explicitly transferred via this non-linguistic information. We define *reliability of cue* (*RC*) to denote how often the listener obtains a correct cue (containing the speaker’s chosen meaning) in an utterance exchange; otherwise, the listener receives a wrong cue (containing an integrated meaning randomly selected in the semantic space and distinct from the speaker’s chosen meaning). For example, if *RC* is 0.6, the listener has a 60% chance of obtaining the correct cue in each round of utterance exchange; otherwise, it obtains a wrong cue. The effect of *RC* on language evolution and the coevolution between language and *RC* levels (reflecting the degree of joint attention) have been systematically discussed in [41].

The listener selects some of its lexical, syntactic, and category rules in its LTM that can interpret the heard utterance as integrated meaning(s). Then, the listener compares the cue’s meaning with the meaning(s) comprehended by its linguistic rules, and sets up candidate sets for comprehension. If the cue’s meaning matches exactly or partially the one interpreted by some linguistic rules, the cue joins those rules to form a candidate set. For example, if some linguistic rules provide an incomplete meaning, “chase〈tiger, #〉”, and the cue says “chase〈tiger, sheep〉”, since the constituents specified by the linguistic rules match those in the cue’s meaning, the cue and these linguistic rules form a candidate set, the meaning of which is “chase〈tiger, sheep〉”. Otherwise, if there is no match between the cue’s meaning and the meaning interpreted by some linguistic rules, the cue itself forms a candidate set. If some linguistic rules also provide a complete interpretation, these rules will form another candidate set. For example, if the linguistic rules offer a complete interpretation, “run〈tiger〉”, but the cue says “fight〈wolf, tiger〉”, then, the cue itself forms a candidate set, the meaning of which is “fight〈wolf, tiger〉”. This set will compete with the set formed by the linguistic rules, the meaning of which is “run〈tiger〉”.

The listener calculates the combined strength of each set:

Combined Strength=Avg(str(LexRule(s)))+Avg(aso(Cats)×str(SynRule(s)))+str(Cue) (2)

Here, for a set without a cue, its combined strength is calculated exactly the same as that in production; for a set having a cue, the cue strength is added to the combined strength. After calculation, the listener chooses the set having the highest combined strength for comprehension.

If the combined strength of the set used by the listener for comprehension exceeds a confidence threshold, the utterance exchange is deemed successful. Then, the listener stores the perceived M-U mapping to its STM, and both the speaker and the listener reward the rules in their chosen sets, by adding a fixed amount to their strengths and association weights, and penalize competing rules in other sets, by deducting the same amount from their strengths and association weights. Otherwise, the utterance exchange is deemed failed. Then, the listener discards the perceived mapping, and both agents penalize the rules in their chosen sets. Widely used in many models of language evolution (e.g. [31,40]), such lateral inhibition adjusting mechanism can lead to conventionalization of linguistic knowledge. Throughout the utterance exchange, there is no direct check whether the speaker's intended meaning matches the listener’s comprehended one. In order to equally treat the linguistic information and non-linguistic information (cue), we set the cue strength equal to the confidence threshold.

For linguistic rules stored in the corresponding LTMs, agents frequently (scaled to the population size) deduct a fixed amount from their strengths and association weights. Then, lexical or syntactic rules having negative strengths are discarded from the corresponding LTM, lexical rules having negative association weights to some categories are removed from those categories, and categories having no lexical members are also discarded from the corresponding LTM.

Table 1 shows the parameter setting for the learning mechanisms and communication (see [63,64] for the discussion of the sensitivity of the simulation results to these parameters).

**Table 1:** Parameter setting for the learning mechanisms and communication scenario.

Parameter	Value
Number of utterance exchange per communication	20
Random creation rate	0.25
Reliability of cue (*RC*)	0.6
Cue strength	0.75
Confidence threshold	0.75
Adjusting amount on rule strengths and association weights in competition	0.1
Adjusting amount on rule strengths and association weights in forgetting	0.01
Frequency of forgetting (scaled to population size)	10
Signalling space size	30

Language origin takes place in this model as follows. At the early stage, to encode salient integrated meanings, agents randomly create holistic expressions. These instances allow recurrent patterns to appear. Based on the general learning mechanisms, agents begin to extract recurrent patterns as compositional rules. Then, the competition between holistic and compositional rules occurs both inside and among idiolects. A holistic rule can only express one integrated meaning, whereas a compositional rule, due to combination, can encode many integrated meanings all involving the constituent(s) encoded by this rule. This makes compositional rules be referred to more frequently than holistic rules in communications. Accordingly, compositional rules gradually win the competition. With more compositional rules being shared by agents, a communal language comprising a set of common lexical rules and consistent word orders originates in the population.

## Theoretical Framework and Simulation Setup

### Theoretical framework

**Fig 3:**
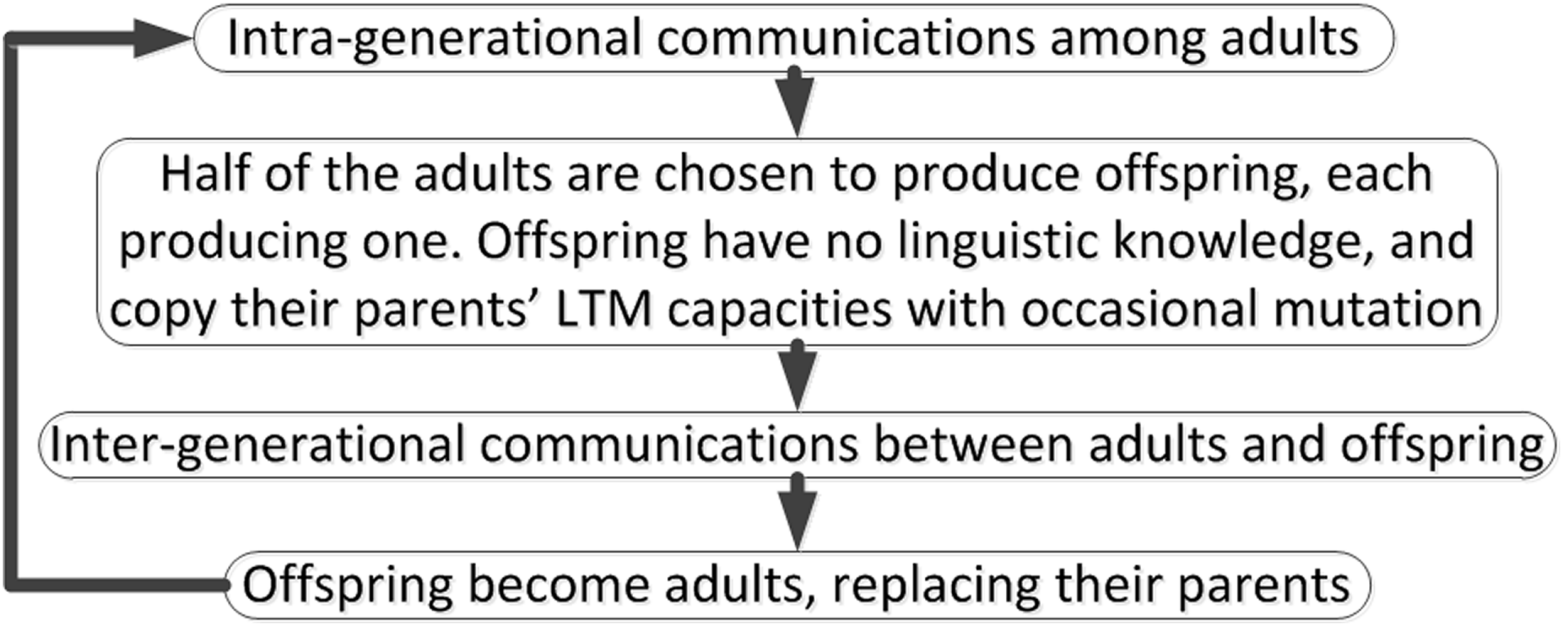
Theoretical framework (adapted from [41]).

The framework involves both *biological transmissions* (offspring copy parents’ LTM capacities with occasional mutation) and *cultural transmissions* (adults talk to each other or to offspring) (see Fig 3). Generation replacement regularly occurs after a fixed number of cultural transmissions. During the replacement, half of the adults are chosen as parents, each producing one offspring. Offspring initially have no linguistic knowledge, and copy their parents’ LTM capacities with occasional mutation. The offspring replace their parents after learning. Such punctuated setting helps explicitly trace the evolution across generations; in reality, however, biological and cultural transmissions are intertwined. In our study, each parent produces one offspring, whereas in [41], each produces two. From the perspective of evolutionary algorithms, these two ways of setting do not induce significantly different results, since the primary factors influencing the simulation results are how to choose parents to reproduce and how to select adults to talk to offspring.

*Communicative success* (*CS*) of an agent indicates the fitness of this agent in the population at each generation. For a particular agent *i*, its *CS* is measured as the mean percentage of meanings that agent *i* can accurately understand (based on acquired linguistic knowledge) when others talk to agent *i*:

$$CS_{i}=\frac{\sum_{j}\text{Number of understandable meanings between agent }j\text{ and agent }i}{\text{Total number of integrated meanings}\times \text{(Number of agents - 1)}}$$ (3)

Note that *CS* can also be defined as the percentage of meanings understandable to others when an agent talks to them, and the simulation results are similar.

Mean *CS* over all adults of a generation is defined as *understanding rate* (*UR*):

$$UR=\frac{1}{\text{Number of agents}}\sum_{i}CS_{i}$$ (4)

A high *UR* shows that agents at that generation can use the evolving language to accurately exchange a large proportion of meanings.

We also measure *Gen* as the ratio of the total number of generations for *UR* to reach a high value (set as 0.8, meaning that most agents can accurately exchange 80% of all meanings):

$$Gen=\frac{\text{Number of generations for evolved }UR\text{ to first exceed }0.8}{\text{Total number of generations}}$$ (5)

Measurement of *Gen* is done after all *UR*s at every sampling points are calculated. If evolved *UR* remains below 0.8 throughout all generations, *Gen* is set to 1.0. *Gen* reflects the efficiency of the origin of a communal language with a high *UR*; the smaller the *Gen*, the more quickly a communal language with a high *UR* can emerge.

As a cultural phenomenon, language use is inseparable from the socio-cultural environment of language speakers; accordingly, cultural selection can cast its influence on language evolution. As an individual behavior, language acquisition and use are also determined by relevant individual cognitive abilities; accordingly, natural selection can also cast its influence on these abilities. The theoretical framework takes account of both natural and cultural selections, and both selections take effect based on individual *CS*. Natural selection chooses adults who can better understand others (having higher *CS*) as parents producing offspring; cultural selection selects adults having higher *CS* as teachers talking to offspring. Here, we only address one type of cultural selection, i.e., manipulating individuals to participate in communications based on their linguistic understandability. There are other types of cultural selection that may affect language and other socio-cultural phenomena [73].

One may argue that since language communications take place in a cultural environment cultural selection should also determine which linguistic rules are kept and which rules are discarded by individuals, and there are many simulations involving exclusively such kind of cultural evolution and leaving out the biological evolution of language to explore whether cultural evolution alone is sufficient to trigger grammar (e.g. [40]). Such argument confuses cultural phenomenon with cultural selection. Language is no doubt a cultural phenomenon. Its evolution proceeds via iterated cultural transmissions among generations of individuals. During transmissions, as shown in other models, given reasonable settings of biological properties (e.g., memory size), agents can develop a communal language by “selecting” linguistic rules frequently and successfully used. Such process also takes place in our model; with or without selections, agents keep choosing and adjusting their available rules to reach mutual understanding. However, the dynamics of language evolution is also subject to constraints from both the cultural aspect (e.g., factors restricting who can interact with whom) and the biological aspect (e.g., factors manipulating individual behaviors during production or perception of linguistic materials), which may exert selective pressures during language evolution. Our framework focuses more on the natural and cultural selections casted by such constraints, and aims to clarify which type of selection plays a more important role in the evolutions of language and related cognitive abilities.

### Simulation Setup

We conduct five sets of simulations based on the theoretical framework. The *NoChange* set contains no cultural and natural selections, nor mutation on LTM capacity. During generation replacement, parents and teachers are randomly chosen, and offspring copy exactly their parents’ LTM capacities. This set of simulations serves as the baseline for the discussion of the coevolution between language and LTM capacities.

The other four sets (*NoNat_NoCul*, without natural and cultural selections; *Nat_NoCul*, with natural selection but without cultural selection; *NoNat_Cul*, without natural selection but with cultural selection and *Nat_Cul*, with both natural and cultural selections) follow a 2×2 factorial design, with natural and cultural selections as two factors each having two levels (in effect or not). When natural selection is in effect, adults with higher *CS* will have higher chances to produce offspring; otherwise, each adult has an equal chance of reproduction. When cultural selection is in effect, adults with higher *CS* will have higher chances to speak to offspring; otherwise, each adult has an equal chance to talk to offspring. In addition, when offspring copy their parents’ LTM capacities, mutation occurs at a constant rate, during which the copied capacity is increased or decreased (with equal chances) with a fixed amount.

In each condition, we conduct 50 simulations. In each simulation, individual LTM capacities and *UR* of the communal language are measured at 201 sampling points evenly distributed throughout 2000 generations. In the *NoChange* set, *Gen* was also measured at the end of each simulation. We adopt a two-way analysis of covariance (ANCOVA [74]) to disentangle the effects of natural and cultural selections on *UR* of the communal language and individual LTM capacities. Statistical analyses are conducted using SPSS v.21.0 (IBM Corp., Armonk, NY, USA). In the analyses, the dependent variable is the mean *UR* or LTM capacity over 50 simulations, the fixed factors are natural and cultural selections, and the covariant is generation (201 sampling points). Using ANCOVA, rather than ANOVA, helps partial out the influence of the covariant on the dependent variable. Apart from ANCOVA, a hierarchical linear model (a.k.a. generalized linear mixed effect models) [75] may also be used to analyze the results. It treats generation as a random factor, and the mean *UR* or mean *LTM* capacities at different generations of a run as repeated measures of those variables. Such model reports similar results to the ANCOVA.

Table 2 lists the parameter setting of most simulations reported in the paper. The population includes 10 agents. Population size correlates with the number of transmissions among adults and the number of transmissions between adults and offspring in each generation: under a bigger population, similar results can be obtained when agents conduct more cultural transmissions per generation. The number of generations is set to 2000, which is sufficient to observe the possible coevolution at a mutation rate 0.05. The mutation rate controls the speed of the adjustment on individual LTM capacity: the smaller the mutation rate, the more generations needed for the coevolution to manifest and *stabilize* (the mean LTM capacity among agents and the mean *UR* of the communal language will not change much across generations). In our study, the mutation rate may not strictly resemble the actual rate in biological transmissions.

**Table 2:** Parameter setting of the simulations.

Parameter	Value
Number of intra-generational transmissions per generation	100
Number of inter-generational transmissions per generation	200
Mutation rate	0.05
Adjusting amount on LTM capacity for lexical rules during mutation	1
Fixed STM capacity	40
Fixed LTM capacities for syntactic rules and categories	40

In most simulations, the semantic space of the evolving language contains 64 meanings, each having identical chances to be produced in communications. Agents in the first generation can only express eight of these meanings using eight shared holistic rules in their LTMs. This limited number of shared rules comprises the preliminary signaling system of early hominins (note that simulations starting with no shared rules show similar results), and the meanings encoded in these rules contain all semantic constituents involved in the total 64 meanings.

New meanings or salient innovations are certainly inevitable during the socio-cultural evolution of language, and formation or acquisition of new semantic concepts also calls for additional individual learning or socio-cultural mechanisms. All these may exert selective pressures on individual memory capacities. However, recklessly including these uncertainties will increase the complexity of the model, or even blur the language-memory coevolution under the natural and/or cultural selections that we consider. Therefore, in the current simulations, the semantic space and semantic constituents are predefined, shared among individuals, and fixed throughout the simulation.

## Simulation Results

### The *NoChange* set

**Fig 4:**
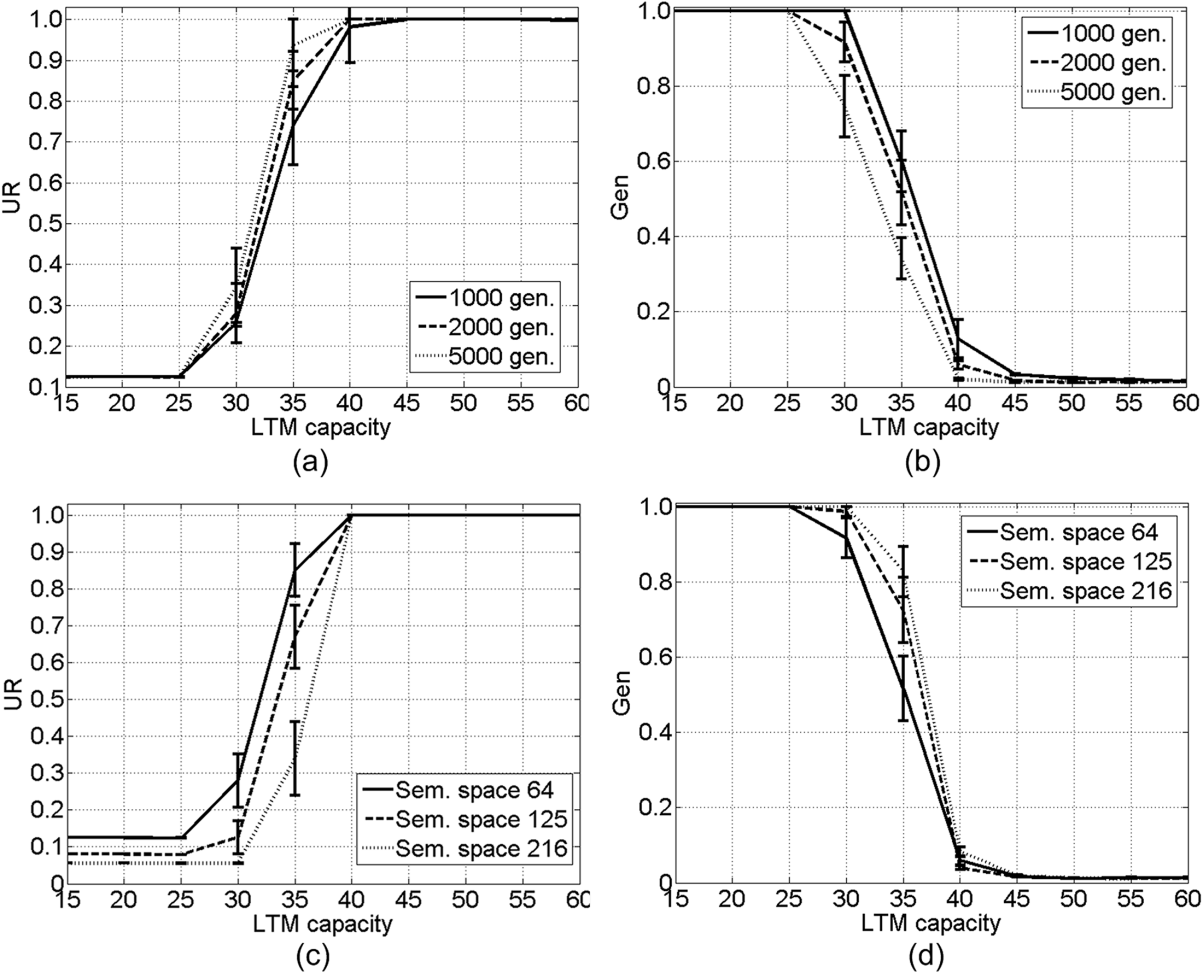
Mean peak-*UR* (the highest *UR* throughout all generations) (a)(c) and *Gen* (b)(d) in the *NoChange* set of simulations. Error bars denote standard errors.

Fig 4(a) and 4(b) show the *UR* and *Gen* in the *NoChange* set of simulations under different initial LTM capacities (from 15 to 60, with step 5). To test the generality of the results, we conduct two additional sets of simulations, both involving no cultural and natural selections nor mutation on LTM capacity. In the first set, the semantic space is fixed at 64, but the number of generations is set to 1000 and 5000, respectively (see Fig 4(a) and 4(b)); in the second set, the number of generations is fixed at 2000, but the size of semantic space is set to 64, 125, and 216, respectively (see Fig 4(c) and 4(d)).

Fig 4 illustrates an inherent correlation between LTM capacity and *UR* (and *Gen*): when the initial LTM capacity is below 30, *UR* remains low and *Gen* equals to 1.0; once it exceeds 30, along with the increase in it, *UR* starts to increase and *Gen* drop; and when it is above 45, *UR* remains high and *Gen* low, indicating that agents can quickly, within a few generations, develop a communal language with high *UR*. This correlation reveals a threshold LTM capacity (around 30), beyond which a communal language with high *UR* can emerge. Such threshold LTM capacity also suggests that without any biological or cultural selection, when the LTM capacity is small, a communal language with high *UR* cannot be triggered solely via cultural evolution during which agents keep selecting frequently and successfully used rules during communications.

One may wonder why the threshold LTM capacity is around 30. Answers to this question lie in the simulated semantic structures and correlations between semantic constituents across expressions, which are beyond the scope of the current study. Per our research question, these simulations confirm the correlation between LTM capacities and the understandability of the emergent language. According to the threshold, we set the initial LTM capacity in the other sets of simulations to two values, one around the threshold (30) and the other above (60), which help reveal whether and how natural and cultural selections cast their effects on the evolutions of language and LTM capacity.

### The sets involving natural and/or cultural selections

In these simulations, the LTM capacity of each agent in the first generation is randomly chosen from a Gaussian distribution, the standard deviation of which is 5 and the mean is 30 (or 60). This setting not only preserves the general characteristic of the population, but also incorporates a certain degree of individual difference.

In the condition of 30 LTM capacity, as for *UR*, the two-way ANCOVA reveals that: (a) natural selection has a significant main effect on *UR* (*F*(1, 40195) = 47979.969, *p* < .001, *η*_*p*_^2^ = .544), whereas cultural selection does not (*F*(1, 40195) = 96.206, *p* = .422, *η*_*p*_^2^ = .000); (b) there is no significant interaction between the two selections (*F*(1, 40195) = 1.297, *p* = .532, *η*_*p*_^2^ = .051); and (c) the covariant (generations) interacts significantly with *UR* (*F*(1, 40195) = 8951.653, *p* < .001, *η*_*p*_^2^ = .182).

**Fig 5:**
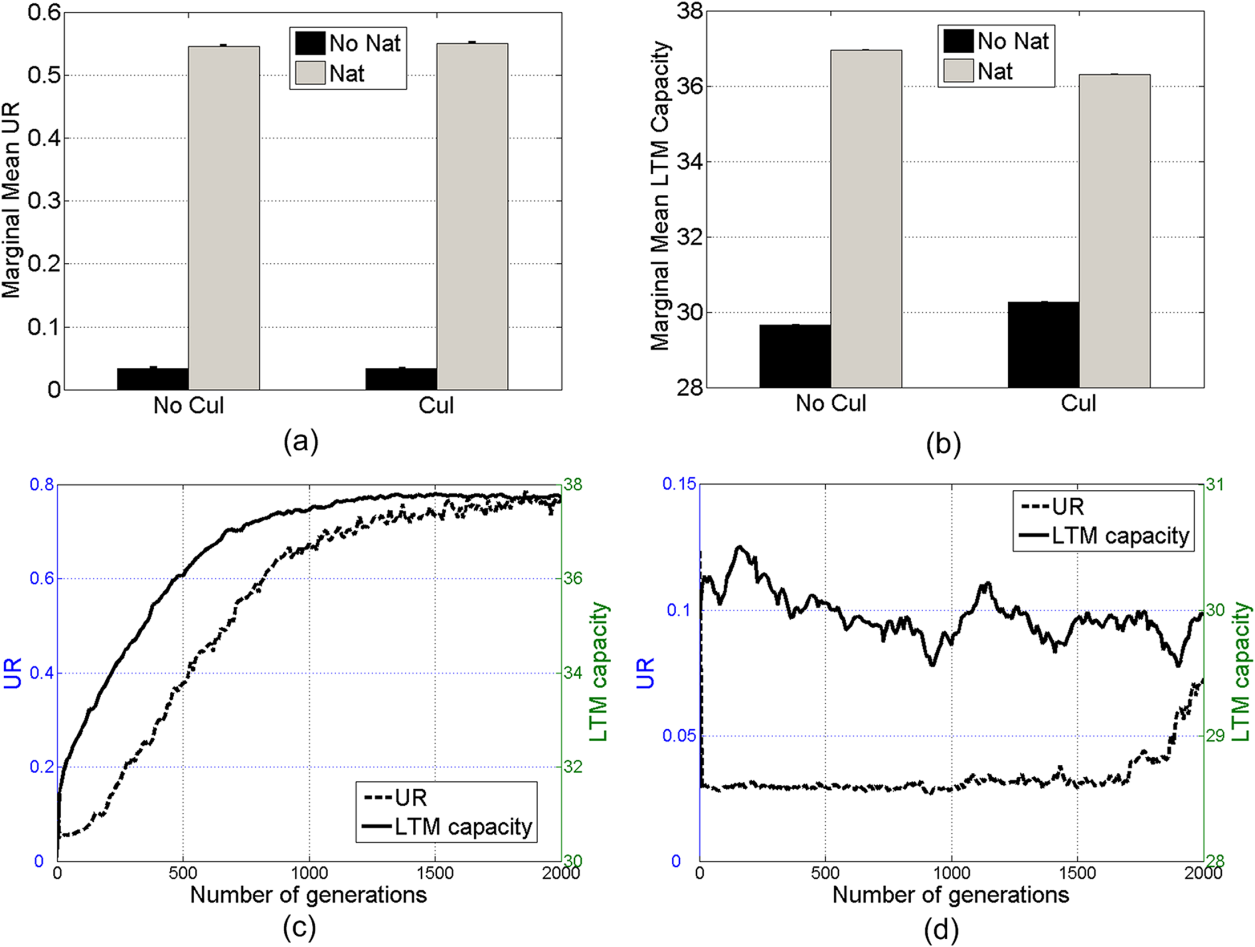
Marginal mean *UR* (a) and LTM capacity (b) in the sets with and without natural (*Nat*) or cultural (*Cul*) selection. Mean *UR* and LTM capacity throughout 2000 generations in the sets with natural selection (c) and without (d). Error bars denote standard errors. Initial LTM capacity is 30.

Natural and cultural selections take effect throughout 2000 generations. Comparing absolute *UR* values at each sampling point would fail to reveal the general effects of these selections. Noting this, we further compare the marginal mean *UR*s (the average *UR* at all sampling points in all simulations) in the four sets of simulations. As shown in Fig 5(a), the marginal *UR* in the sets with natural selection (*Nat_NoCul* and *Nat_Cul*) appears significantly higher than that in the sets without (*NoNat_NoCul* and *NoNat_Cul*), whereas the marginal mean *UR* in the sets with cultural selection (*NoNat_Cul* and *Nat_Cul*) is almost identical to that in the sets without (*NoNat_NoCul* or *Nat_NoCul*). These observations confirm that it is natural selection, rather than cultural selection, that drives the origin of a communal language with high *UR*, which echoes the statistically significant effect of natural selection on *UR*.

As for LTM capacity, the two-way ANCOVA reveals that: (a) natural selection has a significant main effect on the LTM capacity (*F*(1, 40195) = 29360.665, *p* < .001, *η*_*p*_^2^ = .422), whereas cultural selection does not (*F*(1, 40195) = .128, *p* = .721, *η*_*p*_^2^ = .000); (b) there is a significant interaction between the two selections (*F*(1, 40195) = 257.788, *p* < .001, *η*_*p*_^2^ = .006), but the effect size (*η*_*p*_^2^) remains small; and (c) the covariant also interacts significantly with the LTM capacity (*F*(1, 40195) = 1045.447, *p* < .001, *η*_*p*_^2^ = .025).

Similar to *UR*, these results show that the evolution of the LTM capacity is achieved mainly by natural selection, rather than cultural selection. Fig 5(b) shows similar results based on the marginal mean LTM capacity.

Statistical tests reveal that natural selection gradually enhances an initially-low (around the threshold) LTM capacity, along with the origin of a communal language with high *UR*. Such coevolution can also be observed by tracing the mean *UR* and LTM capacity throughout 2000 generations: in the sets with natural selection (see Fig 5(c)), the mean *UR* rises from 0.125 (due to the eight initially-shared rules) to around 0.8, and the mean LTM capacity rises from 30 to around 38; in the sets without natural selection (see Fig 5(d)), however, both the mean *UR* and LTM capacity fluctuate around their initial values throughout generations.

In the condition of 60 LTM capacity, as for *UR*, the two-way ANCOVA shows that: (a) natural selection has a significant, but small effect on *UR* (*F*(1, 40195) = 1915.191, *p* < .001, *η*_*p*_^2^ = .045); (b) cultural selection has a significant, but very small effect on *UR* (*F*(1, 40195) = 16.443, *p* < .001, *η*_*p*_^2^ = .000); (c) there is no significant interaction between the two selections (*F*(1, 40195) = .036, *p* = .850, *η*_*p*_^2^ = .000); and (d) the covariant interacts significantly with *UR*, but the effect is very small (*F*(1, 40195) = 17.230, *p* < .001, *η*_*p*_^2^ = .000).

As for LTM capacity, the two-way ANCOVA shows that: (a) natural selection has a significant, but very small effect on the LTM capacity (*F*(1, 40195) = 95.775, *p* < .001, *η*_*p*_^2^ = .002); (b) cultural selection has a significant, but small effect on the LTM capacity (*F*(1, 40195) = 98.046, *p* < .001, *η*_*p*_^2^ = .002); (c) there is a significant, but small interaction between the two selections (*F*(1, 40195) = 267.043, *p* < .001, *η*_*p*_^2^ = .007); and (d) the covariant has no significant effect (*F*(1, 40195) = 3.482, *p* = .062, *η*_*p*_^2^ = .000).

**Fig 6:**
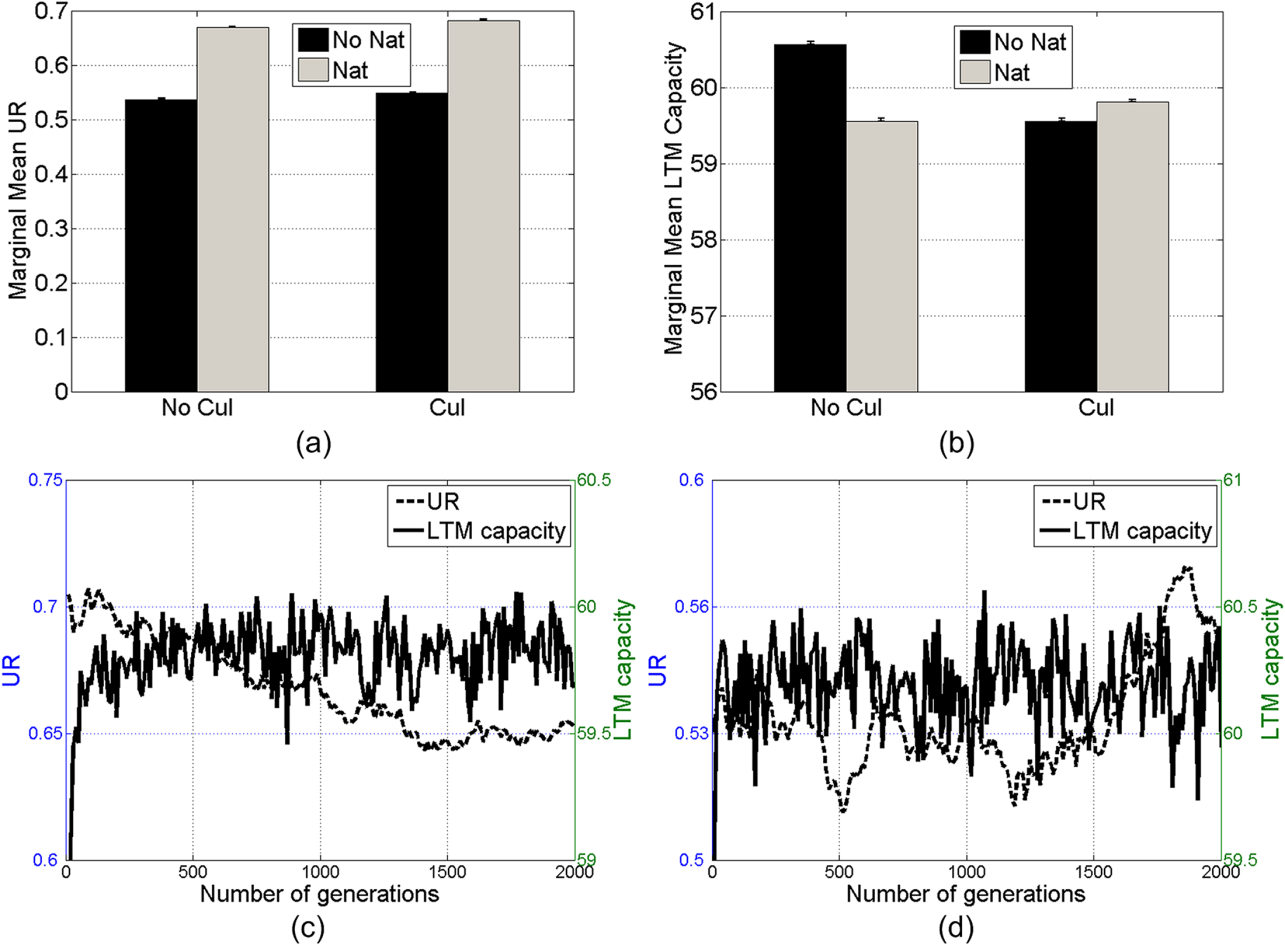
Marginal mean *UR* (a) and LTM capacity (b) in the sets with and without natural (*Nat*) or cultural (*Cul*) selection. Mean *UR* and LTM capacity throughout 2000 generations in the sets with natural selection (c) and without (d). Error bars denote standard errors. Initial LTM capacity is 60.

Fig 6(a) and 6(b) show the marginal mean *UR* and LTM capacity in the four sets of simulations. Differences in these indices remain small; change in *UR* is within 0.15 and change in LTM capacity is within 1. These indicate that when the initial LTM capacity is sufficiently large, both natural and cultural selections will not greatly influence the emergent language and LTM capacity, i.e., the coevolution between language and the LTM capacity as shown in the first condition disappears in the second condition.

Disappearance of the coevolution is also evident by tracing the mean *UR* and LTM capacity throughout 2000 generations. As shown in Fig 6(c) and 6(d), the mean *UR* and LTM capacity fluctuate around certain values, and the small differences of these indices between the sets with and without natural selection reflect the small main effects of natural or cultural selection on these indices. Note that the mean *UR* in Fig 6(c) or 6(d) is lower than 1.0 (complete understanding). This is due to the mutation and individual differences (agents have distinct initial LTM capacities) in these sets of simulations.

To evaluate the generality of these results, we conduct additional simulations under the same initial LTM capacities (30 and 60) but different sizes of the semantic space (125 and 216). These simulations show similar results (see S1 and S2 Figs).

**Fig 7:**
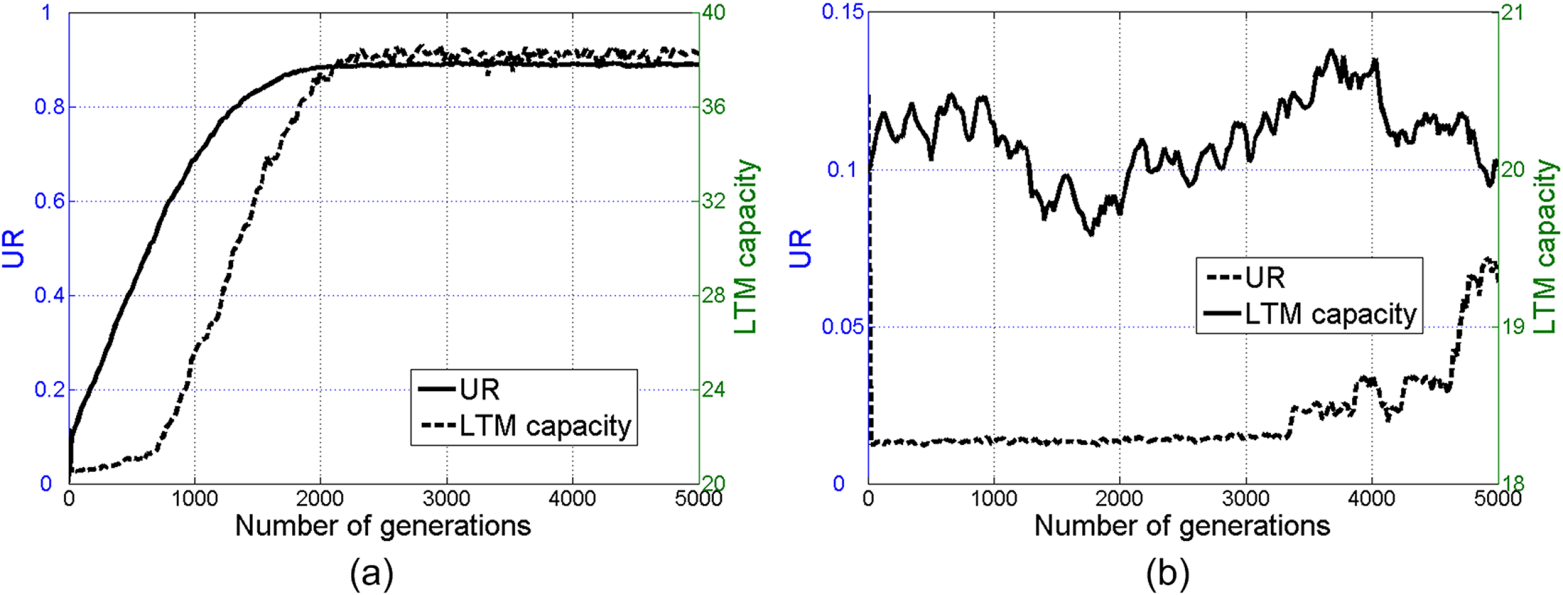
Mean *UR* and LTM capacity throughout 5000 generations in the sets with natural selection (a) and without (b). Initial LTM capacity is 20.

What happens if the initial LTM capacity is further below the threshold? To answer this question, we conduct another set of simulations under an initial LTM capacity of 20, much lower than the threshold shown in Fig 4. To give agents enough time to develop their LTM capacities, we increase the number of generations to 5000. A similar coevolution manifests in these simulations (see Fig 7): with natural selection, an initially-low LTM capacity is gradually enhanced above the threshold and a communal language with high *UR* is efficiently triggered; without natural selection, however, both the mean *UR* and LTM capacity fluctuate around their initial values.

To evaluate the generality of these results, we conduct additional simulations under the same initial LTM capacities (20) but a bigger population (100 agents). Considering the correlation between the population size and the number of cultural transmissions per generation, we set the numbers of intra-generational and inter-generational transmissions to 1000 and 2000, respectively. A similar coevolution also takes place in these simulations (see S3 Fig).

## General Discussions and Conclusions

### Language-memory coevolution

These simulations illustrate a language-memory coevolution during language origin: an initially-low memory capacity is gradually enhanced to better serve the purpose of mutual understanding, and such coevolution disappears once the memory capacity reaches a sufficiently high level such that a communal language with high mutual understandability can efficiently emerge in the population.

As shown in the simulations, the initial LTM capacity (20 or 30), the threshold capacity (around 30), and the sufficient capacity (around 40) resulting from the coevolution are all smaller than the total number of integrated meanings in the semantic space (64 or more). Recall that the initially shared rules among agents are holistic, each encoding one integrated meaning involving an action, action instigator, and/or entity that undergoes the action. If agents keep using such holistic rules to encode meanings, 30, 40, or any value smaller than the total size of the semantic space would not be sufficient. However, checking the lexical rules shared by individuals, we find that along with the coevolution, most shared lexical rules are compositional, each encoding one semantic constitute (an action or an entity that performs or undergoes an action). Compared to the size of the semantic space, the number of semantic constituents is much smaller, and lexical rules encoding such constituents can be fully stored in limited LTM: in the semantic space of 64 meanings, there are only 12 semantic constituents; in the semantic space of 125 meanings, there are only 15 constituents; and in the semantic space of 216 meanings, there are only 18 constituents (note that the integrated meanings in a real language may not be constructed in this way, yet such arbitrary setting of semantic expressions can partially reflect the correlation between integrated meanings and semantic constituents therein). Regulated by consistent grammar (specified by similar syntactic and categorical rules), the small amount of compositional rules can combine to exchange most integrated meanings in the semantic space. Such compositional knowledge reduces the needed LTM capacity, thus making the adjustment on LTM capacity to the minimum and in pace with language origin, and strikes a balance between the huge amount of semantic expressions on the one hand and the limited memory capacity on the other. In addition, the transition from a holistic signaling system to a compositional language, due to individual learning mechanisms such as pattern extraction and sequential learning, is another important outcome of the language-memory coevolution, which also reflects an adaption of language to individual memory capacity.

Statistical analyses suggest that the language-memory coevolution is achieved by natural selection, rather than cultural selection or both. In our study, cultural selection selects adults with higher *CS* to talk to offspring. Even if the chosen adults have sufficient LTM capacities, without natural selection, they would not necessarily have higher chances to reproduce and transfer their memory capacities to offspring. In addition, with both selections in effect, adults having higher *CS* also have higher chances to reproduce, and offspring would learn only from those adults. However, such biased learning may damage mutual understandability in the whole population (see [76] for the collective and isolated effects of various forms of cultural transmission on language evolution). Furthermore, although the coevolution is achieved by natural selection, rather than the cultural selection that we define, cultural transmission is indispensable. By providing individuals with opportunities to develop their idiolects and linguistic knowledge, cultural transmission constructs the niche for natural selection to choose capable individuals based on their linguistic performances [6,17] and thereby cast its effect on the coevolution between language and memory. From this point, our simulations reflect the effect of linguistic culture on human cognition [8,77], and demonstrate that both biological and cultural transmissions are imperative for language evolution [78].

The language-memory coevolution shown in our study is in line with the coevolution between language and joint attention [41], both illustrating that in the context of language origin genetic assimilation can retain and expand communicatively-effective characteristics [77]. Once the LTM capacity or the degree of joint attention becomes associated with language use and communicative success leads to functional advantage (e.g., reproduction opportunities) for capable individuals, under the drive of communicative success, LTM or joint attention can piggyback on language, having its capacity or degree enhanced along with language origin. Nonetheless, there is a difference between the two types of coevolution: when the degree of joint attention is sufficiently high, natural selection helps ratchet this high degree; however, when the LTM capacity is large, both natural and cultural selections stop casting significant influences on the LTM capacity.

Such difference is partially due to the distinct roles of joint attention and LTM capacity in language communications. As for joint attention, it controls whether a common ground can be established so that the listener can receive a cue containing the speaker’s intended meaning. In this situation, cultural selection cannot cast a direct effect on joint attention. Even if many rounds of communications are available, without sufficient common grounds, listeners still fail to grasp a sufficient amount of shared knowledge to understand each other. Therefore, even though the degree of joint attention is high, natural selection remains necessary to preserve this high degree for achieving mutual understandability. As for LTM capacity, it determines whether there is enough space for acquired linguistic knowledge. Given sufficient memory capacities, agents can develop similarly-high degrees of mutual understandability in each generation, thus having roughly-equal chances to be parents (or teachers). In this situation, even without any selection, a high *UR* and not-greatly-changed LTM capacity can be preserved across generations. Therefore, the effects of both natural and cultural selections on the LTM capacity (and *UR*) become implicit.

### Limitations of the current model

“Essentially, all models are wrong, but some are useful.” [79] (p. 242). The adopted language origin model in our study inevitably involves some aspects of simplicity and specificity [37]. For example, similar to most available models of language origin and evolution, our model assumes that agents already have the intention to communicate with each other, and implements a simple way of deducting encoded meanings in exchanged utterances from unreliable environment cues and linguistic rules. There are a bunch of theoretical and experimental explorations on how communicative intention itself could emerge and get detected by humans by means of a set of meta-cognitive abilities (e.g. [26,80]). Although this issue appears to be important for language origin and human cognition, the current model could not address it.

In addition, the model implements a specific set of learning mechanisms to develop and acquire particular types of semantic expressions. With necessary modification, a similar language-memory coevolution may also occur in other models involving other types of processing mechanisms and linguistic expressions. In other words, the language-memory coevolution should not be restricted within the current detailed model.

Furthermore, our simulations define a fixed semantic space. Nonetheless, we can reasonably predict that the coevolution between language and LTM capacity ensures that when linguistic complexity increases (e.g., expressions containing salient constituents or structures become available, thus requiring storing more or new types of linguistic knowledge in memory), the relevant memory capacity will keep adapting accordingly.

Real language is surely much more abundant in semantic expressions and syntactic forms than what are simulated in the current model. Instead of estimating the concrete memory capacities of early hominins (some comparative findings may shed light on this question, e.g. [81]), the foci of our study lie in: whether natural and/or cultural selections can cause adjustment on relevant memory capacity to better serve the purpose of storing linguistic knowledge for mutual understanding; and under what conditions such adjustment manifests or ceases to effect. Findings in these small-scale simulations are informative to the actual coevolution between language and memory capacity under much bigger semantic space and more complex setting.

### Coevolution hypothesis between language and cognition

A common challenge to the coevolution hypothesis between language and cognition is that change in language, which is transmitted mainly via cultural transmission, usually occurs faster than change in socio-cognitive abilities, which is transmitted mainly via biological transmission [29,82,83].

Our study is not subject to this challenge. First of all, neither joint attention nor LTM capacity is language-specific; instead, they are shared by humans and non-human primates, exist prior to language, and naturally participate in general communicative activities. Therefore, it would take a lesser evolutionary step to adopt these competencies for language use than it is to invent these competences to serve this purpose from scratch [16], and genetic assimilation on such general competencies could effectively catch up with the changing language [82].

In addition, both types of coevolution (between language and memory capacity and between language and joint attention) could be most possible and explicit at the early stage of language origin. After coevolution, language is free to vary during cultural evolution, and individuals can keep pace with it using their well-developed cognitive abilities.

Furthermore, so far there lacks decisive evidence on whether the degree of joint attention or the LTM capacity are transmitted genetically (some genetic research shows that a lexicon size is very likely to be inheritable [33]). Neuroimaging studies discover that long-term training can also gradually induce neural or anatomical changes in monkey and human brains, thus influencing their cognitive abilities [84]. Such neural evidence points out that there could be alternative ways than genetic transmissions to adjust the degrees of cognitive abilities. Even though, the degree-difference in joint attention or memory capacity between humans and non-human primates can still be ascribed to the selective pressure of communicative success during cultural transmissions.

The coevolution hypothesis between language and related cognitive abilities advocates that there exists no clear-cut distinction between biological and cultural evolutions (especially during language origin) [6,24], and that human language is *par excellence* a bio-cultural hybrid originating via a continuous coevolution [21,28,85]. These perspectives help revise the dogma that the biological evolution leading to language readiness and the cultural evolution of modern languages must take place at consecutive stages of language evolution [10,86].

To further verify the coevolution hypothesis and better understand the evolutions of language and cognition, we need to keep exploring whether the hypothesis can also interpret the degree differences in other language-related abilities between humans and other animals, and meanwhile, seek more direct evidence in neuroscience, psychology, and animal behavior studies about how these abilities are applied, transmitted, and adjusted in humans and other animals. To meet these challenges, an interdisciplinary approach integrating the knowledge and techniques from not only linguistics but also a variety of relevant disciplines, as exemplified in our study and many others (e.g. [41,87]), has been proved to be the most effective [2,3,9,16,57,78,88,89].

## Supporting Information

S1 Fig Mean *UR* and LTM capacity throughout 2000 generations in the sets with natural selection (a)(c) and without (b)(d). Initial LTM capacity is 30. In (a)(b), the semantic space has 125 meanings. In (c)(d), the semantic space has 216 meanings.
(TIF)

S2 Fig Mean *UR* and LTM capacity throughout 2000 generations in the sets with natural selection (a)(c) and without (b)(d). Initial LTM capacity is 60. In (a)(b), the semantic space has 125 meanings. In (c)(d), the semantic space has 216 meanings.
(TIF)

S3 Fig Mean *UR* and LTM capacity throughout 2000 generations in the sets with natural selection (a) and without (b). The population has 100 agents. Initial LTM capacity is 20.
(TIF)

## References

[1] Botha R, Knight C, eds. (2013) The evolutionary emergence of language: Evidence and inference. Oxford: Oxford University Press 356 p.

[2] Gong T, Shuai L, Wu Y (2013) Multidisciplinary approaches to evolutionary linguistics. Language Sciences 37: 1–13.

[3] Lefebvre C, Comrie B, Cohen H, eds. (2013) New perspectives on the origins of language. Amsterdam: John Benjamins 582 p.

[4] Tallerman M, Gibson KR, eds. (2013) The Oxford handbook of language evolution. Oxford: Oxford University Press 800 p.

[5] Hauser MD, Chomsky N, Fitch WT (2002) The faculty of language: What is it, who has it, and how did it evolve? Science 298: 1569–1579. 12446899 10.1126/science.298.5598.1569

[6] Deacon WT (1997) The symbolic species: The coevolution of language and the brain. New York, NY: W. W. Norton 527 p.

[7] Corballis MC (2002) From hand to mouth: The origins of language. Princeton, NJ: Princeton University Press 257 p.

[8] Tomasello M (2009) Origins of human communication. Cambridge, MA: MIT Press 408 p.

[9] Fitch WT (2010) The evolution of language. Cambridge: Cambridge University Press 610 p.

[10] Arbib M (2013) How the brain got language: The mirror system hypothesis. Oxford: Oxford University Press 413 p.

[11] Chomsky N (1995) The minimalism program. Cambridge, MA: MIT Press 420 p.

[12] Chomsky N (2004) Language and mind: Current thoughts and ancient problems In: Jenkins L, ed. Variation and universals in biolinguistics. Amsterdam: Elsevier pp. 379–405.

[13] Chomsky N (2010) Some simple evo-devo theses: How true might they be for language? In: Larson RK, Déprez V, Yamakido H, eds. The evolution of human language: Biolinguistic perspectives. Cambridge: Cambridge University Press pp. 45–62.

[14] Watumull J, Hauser MD, Roberts IG, Homstein N (2014) On recursion. Frontiers in Psychology 4: article 1017. 10.3389/fpsyg.2013.01017 PMC3884515 24409164

[15] Elman J, Bates E, Johnson MH, Karmiloff-Smith A, Parisi D, Plunkett K (1996) Rethinking innateness: A connectionist perspective on development. Cambridge, MA: MIT Press 447 p.

[16] Hurford JR (2007) The origins of meaning. Oxford: Oxford University Press 388 p.

[17] Hurford JR (1989) Biological evolution of the Saussurean sign as a component of the Language Acquisition Device. Lingua 77: 187–222.

[18] Pinker S, Bloom P (1990) Natural language and natural selection. Behav Brain Sci 13: 707–784.

[19] Thompson JN (1994) The coevolutionary process. Chicago, IL: University of Chicago Press 376 p.

[20] Feldman MW, Laland KN (1996) Gene-culture coevolutionary theory. Trends in Eco Evo 11: 453–457. 10.1016/0169-5347(96)10052-5 21237920

[21] Laland KN, Odling-Smee J, Myles S (2010) How culture shaped the human genome: bring genetics and the human sciences together. Nature Rev Genetics 11: 793–808. 10.1038/nrg2734 20084086

[22] Odling-Smee J (2003) Niche construction: The neglected process in evolution. Princeton, NJ: Princeton University Press 472 p.

[23] Deacon WT (2010) A role for relaxed selection in the evolution of the language capacity. Proc Natl Acad Sci USA 107(suppl 2): 9000–9006. 10.1073/pnas.0914624107 20445088 PMC3024028

[24] Kendal J, Tehrani JJ, Odling-Smee J (2011) Human niche construction in interdisciplinary focus. Phil Trans R Soc B: Biol Sci 366: 785–793. 10.1098/rstb.2010.0306 PMC3048995 21320894

[25] Tomasello M, Carpenter M, Call J, Behne J, Moll H (2005) Understanding and sharing intentions: the origins of cultural cognition. Behav Brain Sci 28: 675–691. 16262930 10.1017/S0140525X05000129

[26] Sperber D, ed. (2000) Metarepresentations: A multidisciplinary perspective. Oxford: Oxford University Press 448 p.

[27] Scott-Phillips TC, Blythe RA, Gardner A, West SA (2012) How do communication systems emerge? Proc R Soc B: Biol Sci 279: 1943–1949. 10.1098/rspb.2011.2181 PMC3311886 22217724

[28] Számadó S, Szathmáry E (2013) Evolutionary biological foundations of the origin of language: The coevolution of language and brain In: Tallerman M, Gibson KR, eds. The Oxford handbook of language evolution. Oxford: Oxford University Press pp. 157–167.

[29] Christiansen MH, Chater N (2008) Language as shaped by the brain. Behav Brain Sci 31: 489–558. 18826669 10.1017/S0140525X08004998

[30] Steels L (2005) The emergence and evolution of linguistic structure: From lexical to grammatical communication systems. Conn Sci 17(3–4): 213–230.

[31] Steels L (2011) Modeling the cultural evolution of language. Phys Life Rev 8(4): 339–356. 10.1016/j.plrev.2011.10.014 22071322

[32] Bishop DVM (2001) Genetic and environmental risks for specific language impairment in children. Phil Trans R Soc London 356: 369–380. 11316485 10.1098/rstb.2000.0770 PMC1088433

[33] Stromswold K (2001) The heritability of language: A review and meta-analysis of twin, adoption, and linkage studies. Language 77: 647–723.

[34] Watkins KE, Dronkers NF, Vargha-Khadem F (2002) Behavioral analysis of an inherited speech and language disorder: comparison with acquired aphasia. Brain 125: 452–464. 11872604 10.1093/brain/awf058

[35] Leonard LB (2014) Specific language impairment across languages. Child Dev Persp 8: 1–5. 10.1111/cdep.12053 PMC3994122 24765105

[36] Wagner K, Reggia JA, Uriagereka J, Wilkinson GS (2003) Progress in the simulation of emergent communication and language. Adap Behav 11(1): 37–69.

[37] Gong T, Shuai L (2013) Computer simulation as a scientific approach in evolutionary linguistics. Language Sciences 40: 12–23.

[38] Batali J (1998) Computational simulations of the emergence of grammar In: Hufrod JR, Studdert-Kennedy M, Knight C, eds. Approaches to the evolution of language: Social and cognitive bases. Cambridge: Cambridge University Press pp. 405–426.

[39] Kirby S (1999) Function, selection, and innateness: The emergence of language universals. Oxford: Oxford University Press 156 p.

[40] Steels L, ed. (2012) Experiments in cultural language evolution. Amsterdam: John Benjamins 306 p.

[41] Gong T, Shuai L (2012) Modeling the coevolution of joint attention and language. Proc R Soc B: Biol Sci 279: 4643–4651. 10.1098/rspb.2012.1431 PMC3479722 22977146

[42] Atkins PWB, Shiffrin RM (1968) Human memory: A proposed system and its control processes In: Spence KW, ed. The psychology of learning and motivation: Advances in research and theory. New York: Academic Press pp. 89–195.

[43] Baddeley AD (2000) The episodic buffer: A new component of working memory? Trends Cog Sci 4: 417–423. 10.1016/s1364-6613(00)01538-2 11058819

[44] Baddeley AD, Hitch GJ (1974) Working memory In: Bower GA, ed. Recent advances in learning motivation. New York: Academic Press pp. 47–90.

[45] Baddeley AD (2003) Working memory and language: An overview. J Comm Dis 36: 189–208. 10.1016/s0021-9924(03)00019-4 12742667

[46] Tulving E (1972) Episodic and semantic memory In: Tulving E, Donaldson W, eds. Organization of memory. New York: Academic Press pp. 381–403.

[47] Baddeley AD, Papagno C, Vallar G (1988) When long-term learning depends on short-term storage. J Mem Lang 27: 586–595.

[48] Atkins PWB, Baddeley AD (1998) Working memory and distributed vocabulary learning. Applied Psycholinguistics 19: 537–552.

[49] Baddeley AD, Gathercole SE, Papagno C (1998) The phonological loop as a language learning device. Psycho Rev 105: 158–173. 10.1037/0033-295x.105.1.158 9450375

[50] Miller GA (1956) The magic number seven, plus or minus two: Some limits on our capacity for processing information. Psycho Rev 63: 81–97. 13310704

[51] Nelson C (2001) The magical number four in short-term memory: A reconsideration of mental storage capacity. Behav Brain Sci 24: 87–173. 11515286 10.1017/s0140525x01003922

[52] Hulme C, Maughan S, Brown GDA (1991) Memory for familiar and unfamiliar words: Evidence for a long-term memory contribution to short-term memory span. J Mem Lang 30: 685–701.

[53] Wood R, Baxer P, Belpaeme T (2011) A review of long term memory in natural and synthetic systems. Adap Behav 20: 81–103.

[54] Van der Lely HKJ, Howard D (1993) Children with specific language impairment: Linguistic impairment or short-term memory deficit? J Speech Hearing Res 36: 1193–1207. 8114487 10.1044/jshr.3606.1193

[55] Gathercole SE, Baddeley AD (1990) Phonological memory deficits in language disordered children: Is there a causal connection? J Mem Lang 29: 336–360.

[56] Lum JAG, Gelgic C, Conti-Ramsden G (2010) Procedural and declarative memory in children with and without specific language impairment. Intl J Lang Comm Dis 45: 96–107. 10.3109/13682820902752285 PMC2826154 19900077

[57] Võsu E, Kõresaar E, Kuutma K (2008) Mediation of memory: Towards transdisciplinary perspectives in current memory studies. TRAMES 12: 243–263.

[58] Boyd R, Richerson PJ (2010) Why possibly language evolved. Biolinguistics 4: 289–306.

[59] Mesoudi A, McElligott AG, Adger D (2011) Introduction: integrating genetic and cultural evolutionary approaches to language. Human Biol 83: 141–151. 10.3378/027.083.0201 21615283

[60] Baronchelli A, Felici M, Loreto L, Caglioti E, Steels L (2006) Sharp transition towards shared vocabularies in multi-agent systems. J Stat Mech: P06014.

[61] Puglisi A, Baronchelli A, Loreto V (2008) Cultural route to the emergence of linguistic categories. Proc Natl Acad Sci USA 105(23): 7936–7940. 10.1073/pnas.0802485105 18523014 PMC2430341

[62] Smith K, Kirby S, Brighton H (2003) Iterated learning: A framework for the emergence of language. Artificial Life 9(4): 371–386. 14761257 10.1162/106454603322694825

[63] Gong T (2009) Computational simulation in evolutionary linguistics: A study on language emergence. Taipei: Academia Sinica 354 p.

[64] Gong T (2011) Simulating the coevolution of compositionality and word order regularity. Inter Stud 12: 63–106.

[65] Tomasello M (2003) Constructing a language: A usage-based theory of language acquisition. Cambridge, MA: Harvard University Press 388 p.

[66] Arbib M (2008) Holophrasis and the protolanguage spectrum. Inter Stud 9: 154–168.

[67] Wray A (2008) Formulaic language: Pushing the boundaries. Oxford: Oxford University Press 305 p.

[68] Elman JL (1990) Finding structure in time. Conn Sci 14(2): 179–211.

[69] Christiansen MH, Chater N (1999) Toward a connectionist model of recursion in human linguistic performance. Cog Sci 23: 157–203.

[70] Bod R (2009) From exemplar to grammar: A probabilistic analogy-based model of language learning. Cog Sci 31: 752–793. 10.1111/j.1551-6709.2009.01031.x 21585486

[71] Bybee J (2006) From usage to grammar: The mind’s response to repetition. Language 82: 711–733.

[72] Arnon I, Snider N (2010) More than words: Frequency effects for multi-word phrases. J Mem Lang 62: 67–82.

[73] Richerson PJ, Christiansen MH, eds. (2013) Cultural evolution: Society, technology, language, and religion. Cambridge, MA: MIT Press 485 p.

[74] Field A (2009) Discovering statistics using SPSS (3rd ed.) London: SAGE 821 p.

[75] McCullagh P, Nelder JA (1989) Generalized linear models (2nd ed.) Chapman & Hall/CRC Press 511 p.

[76] Gong T (2010) Exploring the roles of horizontal, vertical, and oblique transmissions in language evolution. Adap Behav 18: 356–376.

[77] Tomasello M (2011) Human culture in evolutionary perspective In: Gelfand M, ed. Advances in culture and psychology. Oxford: Oxford University Press pp. 5–52.

[78] Hurford JR (2012) The origins of grammar. Oxford: Oxford University Press 791 p.

[79] Box GEP, Draper NR (1987) Empirical model-building and response surfaces. New York: Wiley 669 p.

[80] Scott-Phillips TC, Kirby S, Ritchie GRS (2009) Signaling signalhood and the emergence of communication. Cognition 113: 226–233. 10.1016/j.cognition.2009.08.009 19740461

[81] Fagot J, Cook RG (2006) Evidence for large long-term memory capacities in baboons and pigeons and its implications for learning and the evolution of cognition. Proc Natl Acad Sci USA 103(46): 17564–17567. 17088563 10.1073/pnas.0605184103 PMC1634836

[82] Chater N, Reali F, Christiansen MH (2009) Restrictions on biological adaptation in language evolution. Proc Natl Acad Sci USA 106: 1015–1020. 10.1073/pnas.0807191106 19164588 PMC2633574

[83] Christiansen MH, Reali F, Chater N (2011) Biological adaptations for functional features of language in the face of cultural evolution. Human Biol 83: 247–259. 10.3378/027.083.0206 21615288

[84] Scholz J, Klein MC, Behrens TEJ, Johansen-Berg H (2009) Training induces changes in white-matter architecture. Nature Neurosci 12: 1367–1368. 19820707 10.1038/nn.2412 PMC2770457

[85] Levinson SC, Dediu D (2013) The interplay of genetic and cultural factors in ongoing language evolution In: Richerson PJ, Christiansen MH, eds. Cultural evolution: Society, technology, language, and religion. Cambridge, MA: MIT Press pp. 219–232.

[86] Berwick RC, Friederici AD, Chomsky N, Bolhuis JJ (2013) Evolution, brain, and the nature of language. Trends Cog Sci 17: 91–100. 10.1016/j.tics.2012.12.002 23313359

[87] Zhang M, Gong T (2013) Principles of parametric estimation in modeling language competition. Proc Natl Acad Sci USA 110(24): 9698–9703. 10.1073/pnas.1303108110 23716678 PMC3683775

[88] Gong T, Yang R, Zhang C, Umberto A (2010) Review of the summer institute in cognitive sciences 2010: The origins of language. Biolinguistics 44: 385–402.

[89] Umberto A, Gong T (2015) Review of New perspectives on the origins of language. Ed. by Lefebvre Claire, Comrie Bernard, and Cohen Henri. (Studies in language companion series 144.). Language 91(1): 256–260.

