# Supplementary figures and images for: Modeling Coevolution between Language and Memory Capacity during Language Origin
Source: PLoS One. 2015 Nov 6;10(11):e0142281. doi: 10.1371/journal.pone.0142281 (PMC4636343; doi:10.1371/journal.pone.0142281)

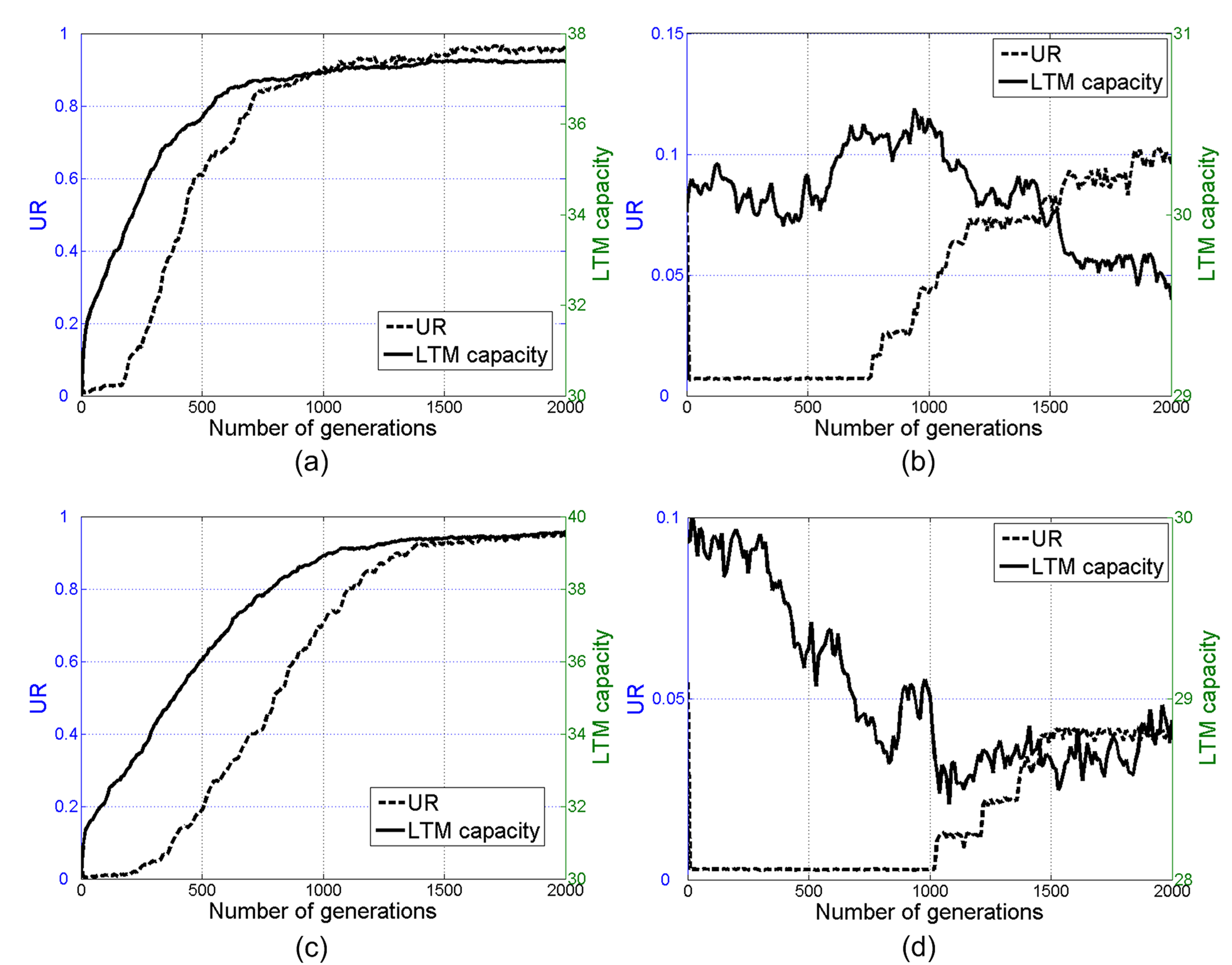

Supplement: S1 Fig — Initial LTM capacity is 30. In (a)(b), the semantic space has 125 meanings. In (c)(d), the semantic space has 216 meanings. (TIF) [file pone.0142281.s001.tif]

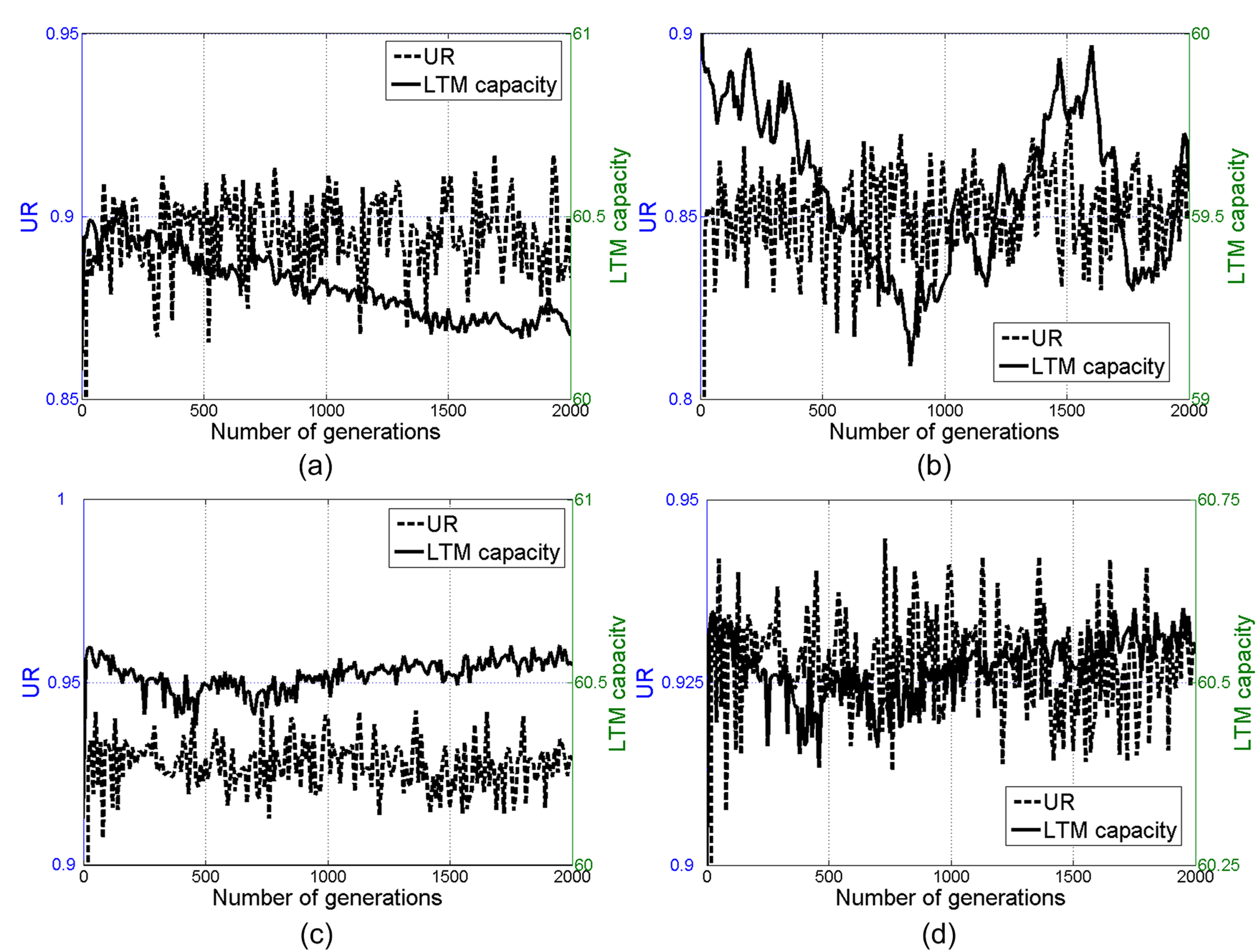

Supplement: S2 Fig — Initial LTM capacity is 60. In (a)(b), the semantic space has 125 meanings. In (c)(d), the semantic space has 216 meanings. (TIF) [file pone.0142281.s002.tif]

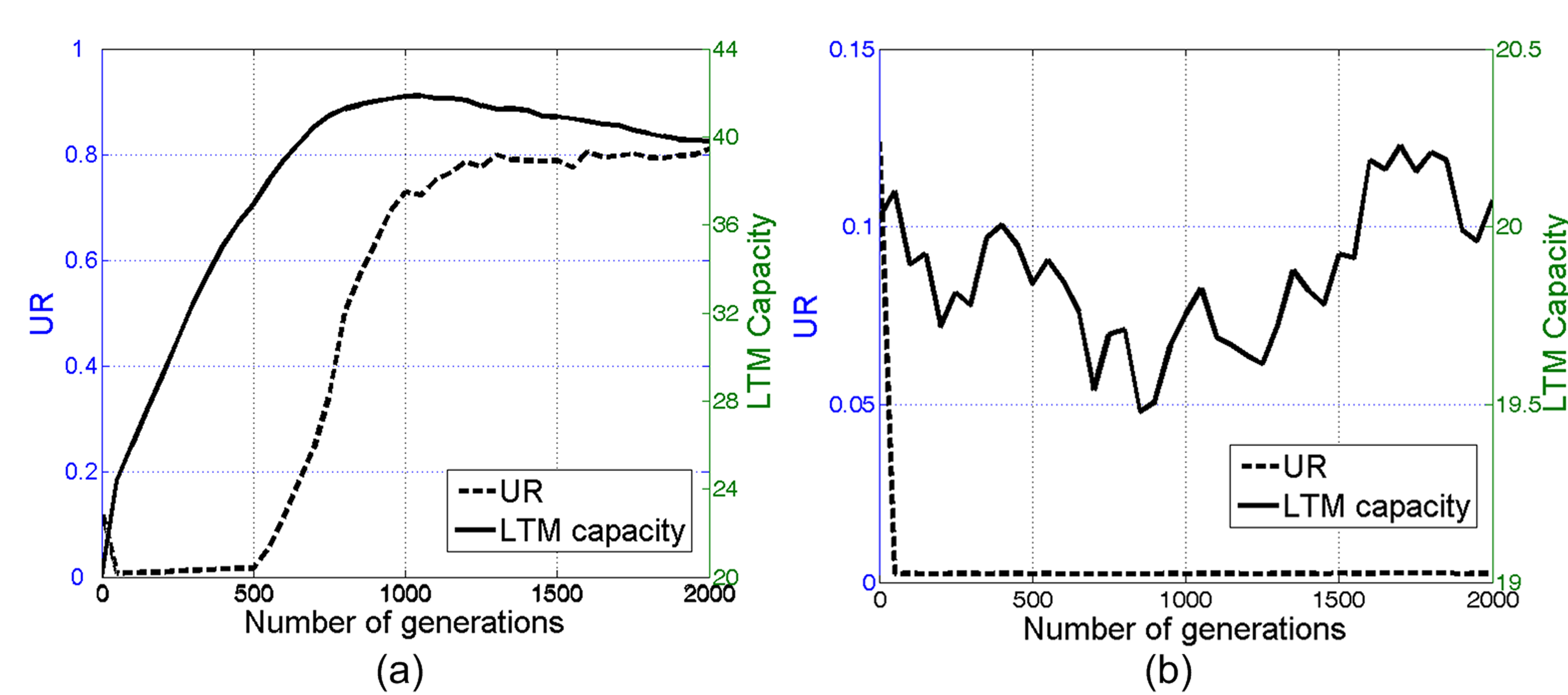

Supplement: S3 Fig — The population has 100 agents. Initial LTM capacity is 20. (TIF) [file pone.0142281.s003.tif]
